# CRISPR/Cas9-mediated targeted mutagenesis in Japanese cedar (*Cryptomeria japonica* D. Don)

**DOI:** 10.1038/s41598-021-95547-w

**Published:** 2021-08-10

**Authors:** Yoshihiko Nanasato, Masafumi Mikami, Norihiro Futamura, Masaki Endo, Mitsuru Nishiguchi, Yasunori Ohmiya, Ken-ichi Konagaya, Toru Taniguchi

**Affiliations:** 1 Forest Bio-Research Center, Forestry and Forest Products Research Institute, 3809-1 Ishi, Juo, Hitachi, Ibaraki 319-1301 Japan; 2 Graduate School of Nanobioscience, Yokohama City University, 22-2 Seto, Yokohama, Kanagawa 236-0027 Japan; 3 Plant Genome Engineering Research Unit, Institute of Agrobiological Sciences, National Agriculture and Food Research Organization, 1-2 Owashi, Tsukuba, Ibaraki 305-8634 Japan; 4 Department of Forest Molecular Genetics and Biotechnology, Forestry and Forest Products Research Institute, 1 Matsunosato, Tsukuba, Ibaraki 305-8687 Japan; 5 Kihara Institute for Biological Research, Yokohama City University, 641-12 Maioka-cho, Yokohama, Kanagawa 244-0813 Japan; 6 Present Address: Tohoku Regional Office, Forest Tree Breeding Center, Forestry and Forest Products Research Institute, 95 Osaki, Takizawa, Iwate 020-0621 Japan

**Keywords:** Plant biotechnology, Plant breeding

## Abstract

*Cryptomeria japonica* (Japanese cedar or sugi) is one of the most important coniferous tree species in Japan and breeding programs for this species have been launched since 1950s. Genome editing technology can be used to shorten the breeding period. In this study, we performed targeted mutagenesis using the CRISPR/Cas9 system in *C. japonica*. First, the CRISPR/Cas9 system was tested using green fluorescent protein (GFP)-expressing transgenic embryogenic tissue lines. Knock-out efficiency of GFP ranged from 3.1 to 41.4% depending on U6 promoters and target sequences. The GFP knock-out region was mottled in many lines, indicating genome editing in individual cells. However, in 101 of 102 mutated individuals (> 99%) from 6 GFP knock-out lines, embryos had a single mutation pattern. Next, we knocked out the endogenous *C. japonica* magnesium chelatase subunit I (*CjChlI*) gene using two guide RNA targets. Green, pale green, and albino phenotypes were obtained in the gene-edited cell lines. Sequence analysis revealed random deletions, insertions, and replacements in the target region. Thus, targeted mutagenesis using the CRISPR/Cas9 system can be used to modify the *C. japonica* genome.

## Introduction

*Cryptomeria japonica* D. Don (Japanese cedar or sugi) is an evergreen conifer, which belongs to the cypress family (Cupressaceae) and is distributed across Japan. Outside Japan, *C. japonica* var. *sinensis* (formerly *C. fortunei*) is thought to be naturally distributed in southeast China^1^. *C. japonica* is a commercially important tree in Japan and has been used since ancient times as for timber and to produce household items, etc. because of its high productivity and utility. *C. japonica* cultivation began more than 500 years ago in some areas of Japan^2^. A breeding program has been in progress since 1950s for screening plus-trees, and thus far, more than 3500 plus *C. japonica* tree clones have been selected^3^. *C. japonica* was extensively planted after WWII because of the government's efforts to restore devastated forests. Consequently, *C. japonica* comprises 44% of the planted forest area in Japan, which covers approximately 11% of the land in Japan^4^. *C. japonica* pollinosis has become a severe public health problem in Japan due to the widespread distribution of *C. japonica* pollen^5^. Development of “male-sterile” *C. japonica* trees is a breeding target in Japan. Studies for identifying genes involved in pollen development are underway^6,7^. Four male-sterility loci, namely *MS1*, *MS2*, *MS3*, and *MS4*, have been identified from 23 male-sterile *C. japonica* lines^8^, and Hasegawa et al. elucidated a causative male-sterile gene at the *MS1* locus^9^. Additionally, SNP markers for distinguishing *MS1*-derived male-sterile plants have been developed^10^. Recently, an efficient technique for producing male-sterile *C. japonica* plants using somatic embryogenesis was reported^11^.

To obtain the desired trait through gene modification in a short time, programmable nuclease-mediated targeted mutagenesis is the option of choice and several methods, such as zinc-finger nuclease (ZNF), transcription activator-like effector nuclease (TALEN), and clustered regularly interspaced short palindromic repeat (CRISPR)/CRISPR-associated protein 9 system (CRISPR/Cas9), have been developed. The use of CRISPR/Cas9 system, first reported in 2012^12^, has exploded because of its efficiency and simplicity, and has been applied to at least 45 plant genera^13^. The CRSIPR/Cas9 system has been improved by expanding the targeting range of Cas9^14,15^ and developing smaller Cas proteins^16,17^. In addition, genome editing systems based on the CRISPR/Cas9 system without involving double-strand breaks, such as base editing^18,19^ and prime editing^20^ have been reported. Among woody plants, CRISPR/Cas9-mediated targeted mutagenesis in *Populus*, which is a model woody species, was first reported in 2015^21,22^. However, the use of genome editing in conifers remains unexplored.

In this study, we demonstrated CRISPR/Cas9-mediated targeted mutagenesis in *C. japonica* through *Agrobacterium*-mediated transformation. First, we targeted the exogenous green fluorescent protein (GFP) gene, which was introduced into the cell mass of embryonic tissue, after examining various promoters to construct an efficient targeted mutagenesis system in *C. japonica*. Targeted *GFP* knock-out was observed in transgenic lines at a certain rate with the CRISPR/Cas9 expression vectors. Then, as a case study, we attempted to disrupt an endogenous gene, namely magnesium chelatase subunit I (*CjChlI*) that is required for chlorophyll biosynthesis^23,24^. Disruption of this gene results in an albino or pale green phenotypes, which readily confirms CRISPR/Cas9-mediated targeted mutagenesis. We used a modified vector and obtained *CjChlI* biallelic mutant lines. Thus, the CRISPR/Cas9 system is efficient in *C. japonica*. We discuss future perspectives in genome editing in conifers.

## Materials and methods

This study was conducted at Forest Bio-Research Center, Forestry and Forest Products Research Institute, Ibaraki, Japan during the years from 2015 to 2020 with the relevant institutional, national, and international guidelines and legislation.

### Plant material

An embryogenic tissue (ET) cell line #13-8-12 was initiated using megagametophyte explants of *C. japonica*^25^. The ET was maintained in the dark at 25 °C in solid 1/2MD medium^26^ and subcultured on fresh media after a 2-week interval.

### Vector construction

**Figure 1:**
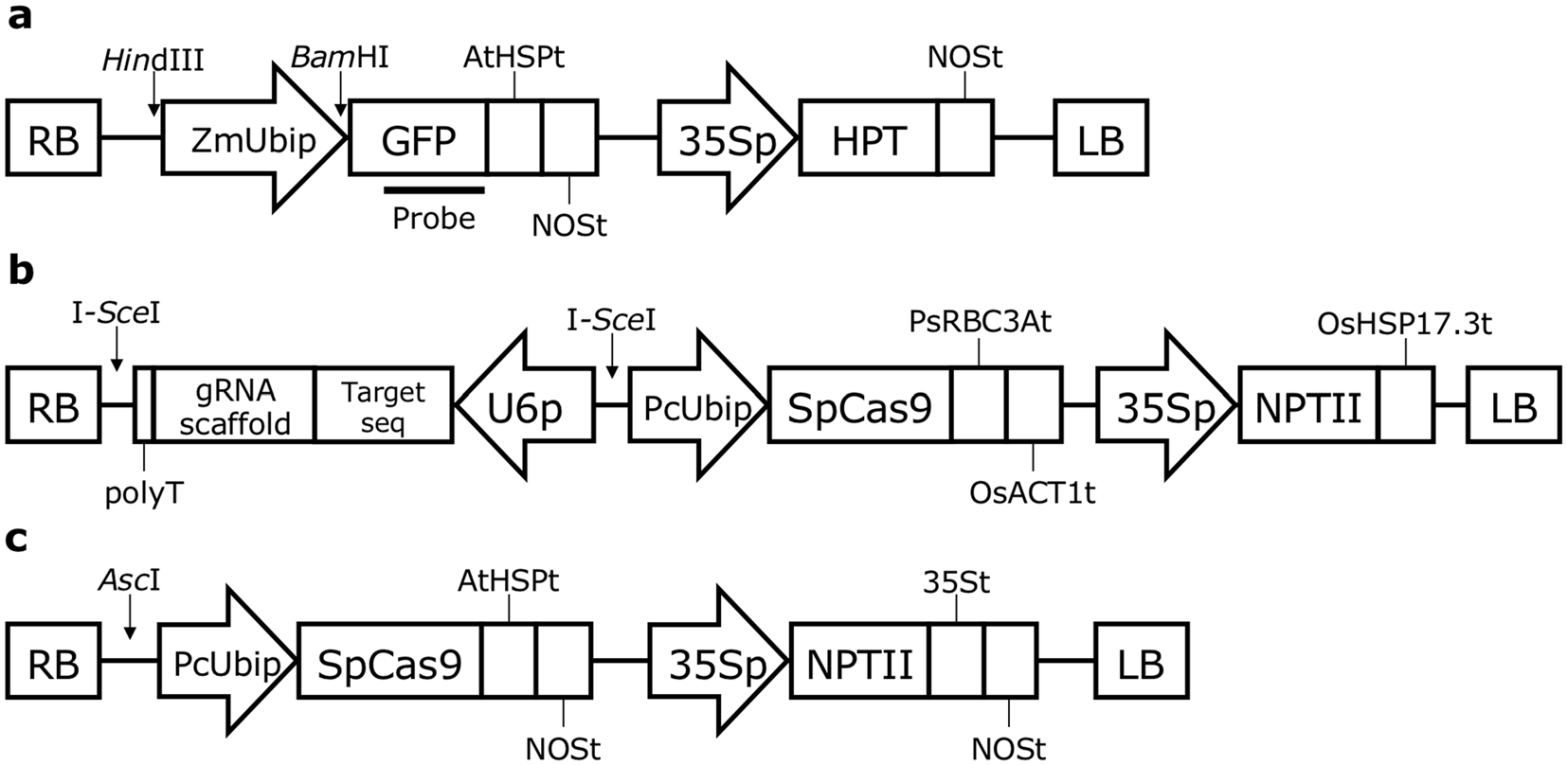
Schematic diagram of the T-DNA region of the GFP expression vector [pZmUbi-GFP-Dt, (**a**)], the CRISPR/Cas9 expression vector [pZK_FFCas9 vector with a gRNA expression cassette, (**b**)], and the modified CRISPR/Cas9 expression vector [pCRG-SpCas9, (**c**)]. HindIII and BamHI sites and the DNA fragment used as the probe for Southern blot analysis are indicated in (**a**). RB, right border; ZmUbip, ubiquitin promoter from *Zea mays*, AtHSPt, heat shock protein terminator from *Arabidopsis thaliana*; NOSt, nopaline synthase terminator; 35Sp, cauliflower mosaic virus 35S promoter; HPT, hygromycin phosphotransferase; LB, left border; U6p, U6 promoter; PcUbip, ubiquitin promoter from *Petroselinum crispum*; SpCas9, Cas9 from *Streptococcus pyogenes* (codon-optimized for *A. thaliana*); PsRBC3At, RBC3A terminator from *Pisum sativum*; OsACT1t, actin-1 terminator from *Oryza sativa*; NPTII, neomycin phosphotransferase; OsHSP17.3t, heat shock protein 17.3 terminator from *O. sativa*.

The GFP expression vector pZmUbi-GFP-Dt (Fig. 1a) was constructed based on pUbiP-sGFP/HygR^25^. In short, the *NPTII* expression cassette was removed from pUbiP-sGFP/HygR using *Pme*I and *Cla*I followed by blunting with the KOD Plus polymerase (Toyobo, Osaka, Japan) and self-ligation using T4 DNA ligase (New England Biolabs, Ipswich, MA). The heat shock protein terminator from *Arabidopsis thaliana*^27^ was inserted downstream of GFP using In-Fusion cloning (In-Fusion HD Cloning Kit, Takara Bio Inc., Shiga, Japan). pZK_FFCas9 (Fig. 1b) was constructed according to Mikami et al.^28^. Briefly, the PcUbi::SpCas9::PsBCB3At fragment from pDeCas9^29^ and the OsAct1t::35S::NPTII::OsHSP17.3t fragment from pE(L3-L2)^28^ were cloned into pZK^30^ using In-Fusion cloning. The gRNA expression cassette driven by the *A. thaliana* U6-26 promoter, derived from pEn-Chimera^29^, was cloned into the multiple-cloning site of pUC19. To use the OsU6 promoter, pUC6gRNA^31^ was used. U6 promoters from *C. japonica* were replaced with the OsU6 promoter in pUC6gRNA (described in Supplementary Information). All oligonucleotides used in this study are shown in Supplementary Table S1. Oligonucleotide pairs for target GFP sequences (p#84 and p#85 for target #1, p#86 and p#87 for target #2_rev, and p#88 and p#89 for target #3_rev) (Table 1, Supplementary Table S1) were annealed, and the resulting fragment was inserted into *Bbs*I sites between the U6 promoter and gRNA scaffold sequence of the arbitrary gRNA expression cassette. The gRNA expression cassette was inserted into the I-*Sce*I site between the right border and the ubiquitin promoter from *Petroselinum crispum* (PcUbi)^32^ in pZK_FFCas9, resulting in the all-in-one CRISPR/Cas9 vector (Fig. 1b). For targeted mutagenesis of endogenous *CjChlI*, the modified CRISPR/Cas9 vector, namely pCRG-SpCas9 (Fig. 1c), was constructed. Briefly, the HSP terminator from *A. thaliana* and NOS terminator were tandemly inserted downstream of FFCas9. The gRNA expression cassette was inserted into the *Asc*I site between the right border and PcUbi in the pCRG-SpCas9 vector through overlapping PCR^33^. The detailed method for vector construction is described in Supplementary Information.

**Table 1:** Target sequence of GFP.

Target	Sequence (5′–3′)	References
#1	GTGAACCGCATCGAGCTGAAGGG	Mali et al.^38^
#2_rev	CCTACGGCGTGCAGTGCTTCAGC	Jacobs et al.^39^
#3_rev	CCACCGGCAAGCTGCCCGTGCCC	Fu et al.^37^

### Transformation of *C. japonica*

ET cell lines expressing GFP were generated through the *Agrobacterium*-mediated transformation of pZmUbi-GFP-Dt/HygR^26,34^. After screening with 5 mg/L hygromycin, lines with a strong GFP signal and a single-copy insertion (named N4) were selected through Southern blot analysis (Supplementary Fig. S1). The N4 line was transformed with pZK_FFCas9 vectors containing various gRNA expression cassettes. Transformed cell lines were selected on 1/2 MD medium containing 10 mg/L meropenem and 25 mg/L kanamycin. Transformed cell lines were maintained at 25 °C under dark. Somatic embryogenesis and germination were performed as described^26^.

### GFP visualization

After screening for 2–4 months in the selection medium, independent lines were isolated and considered a single event. GFP fluorescence of independent lines was evaluated using the MS FLIII fluorescence stereomicroscope (Leica microsystems, Wetzlar, Germany) with a GFP Plus filter system (excitation filter 480/40 nm, emission filter 510 nm). GFP signal was imaged using the DC300 F digital camera system (Leica microsystems).

### Detection of mutations in genomic DNA

Genomic DNA was extracted from the cell mass, the aerial part, or the root using the DNAs-ici!-P kit (Rizo inc. Tsukuba, Japan) or “One-step method” according to the instructions of the KOD FX Neo kit (Toyobo, Osaka, Japan). Target regions were amplified using the KOD-Plus-Neo kit (Toyobo) or KOD FX neo kit (Toyobo). The GFP expression cassette was amplified using the p#113_f and p#49_r primer pair (Supplementary Table S1). PCR conditions were as follows: 94 °C for 2 min; 30–35 cycles at 98 °C for 10 s, 58 °C for 30 s, and 68 °C for 30 s; and final extension at 68 °C for 7 min. PCR products were sequenced directly using p#89_r for GFP target #1 and p#87_r for GFP target #2_rev and #3_rev. For cell lines with multiple indel patterns, PCR products were cloned into pCRII using the Zero-blunt PCR TOPO kit (Invitrogen, Waltham, Massachusetts). Sequence analysis was performed for each clone. To analyze mutation patterns of *CjChlI*-targeted mutagenesis individuals, a fragment containing the target sequence was amplified using primers p#468_f and p#648_r (Supplementary Table S1). PCR conditions were as follows: 94 °C for 2 min; 40 cycles at 98 °C for 10 s, 65 °C for 30 s, and 68 °C for 20 s; and final extension at 68 °C for 7 min. PCR products were cloned into pCRII using the Zero-blunt PCR TOPO kit, and then, sequenced using M13 forward or M13 reverse primers.

### Southern blot analysis

Genomic DNA was isolated from ETs and young needles using Nucleon PhytoPure (Cytiva, Tokyo, Japan). DIG-labeled PCR probes were amplified using p#89_f and p#301_r primers for GFP and #466_f and #501_r primers for *CjChlI* (Supplementary Table S1), according to the manufacturer's instructions (PCR DIG Probe Synthesis Kit, Roche Diagnostics, Mannheim, Germany). Hybridized probes were detected using anti-Dioxigenin-AP (Roche Diagnostics) on an optical imaging system (Fusion Solo 4M, Vilber Lourmat, Marne la Vallee, France).

### Heteroduplex mobility assay

A 215 bp fragment containing the target sites of *CjChlI* was amplified using primers p#468_f and p#469_r (Supplementary Table S1). PCR amplification was performed under the following conditions: 94 °C for 2 min; 40 cycles at 98 °C for 10 s, 65 °C for 30 s, and 68 °C for 20 s; and final extension at 68 °C for 7 min. To ensure full heteroduplex formation, a denaturation and re-annealing procedure was performed on the PCR products as follows: 95 °C for 5 min; 95–85 °C, ramping at − 2 °C/s; and 85–25 °C at − 0.1 °C. Reannealed products were analyzed using a microchip electrophoresis system (MCE-202 MultiNA, Shimazu, Kyoto, Japan) with the DNA-500 reagent kit (Shimazu).

### Statistical analysis

Pairwise multiple comparison of proportions was performed using Tukey’s multiple comparison test conducted in R (https://www.R-project.org/). Significant results were assumed for P < 0.05.

## Results

### Knock-out of a reporter GFP transgene using the CRISPR/Cas9 system with various U6 promoters

**Figure 2:**
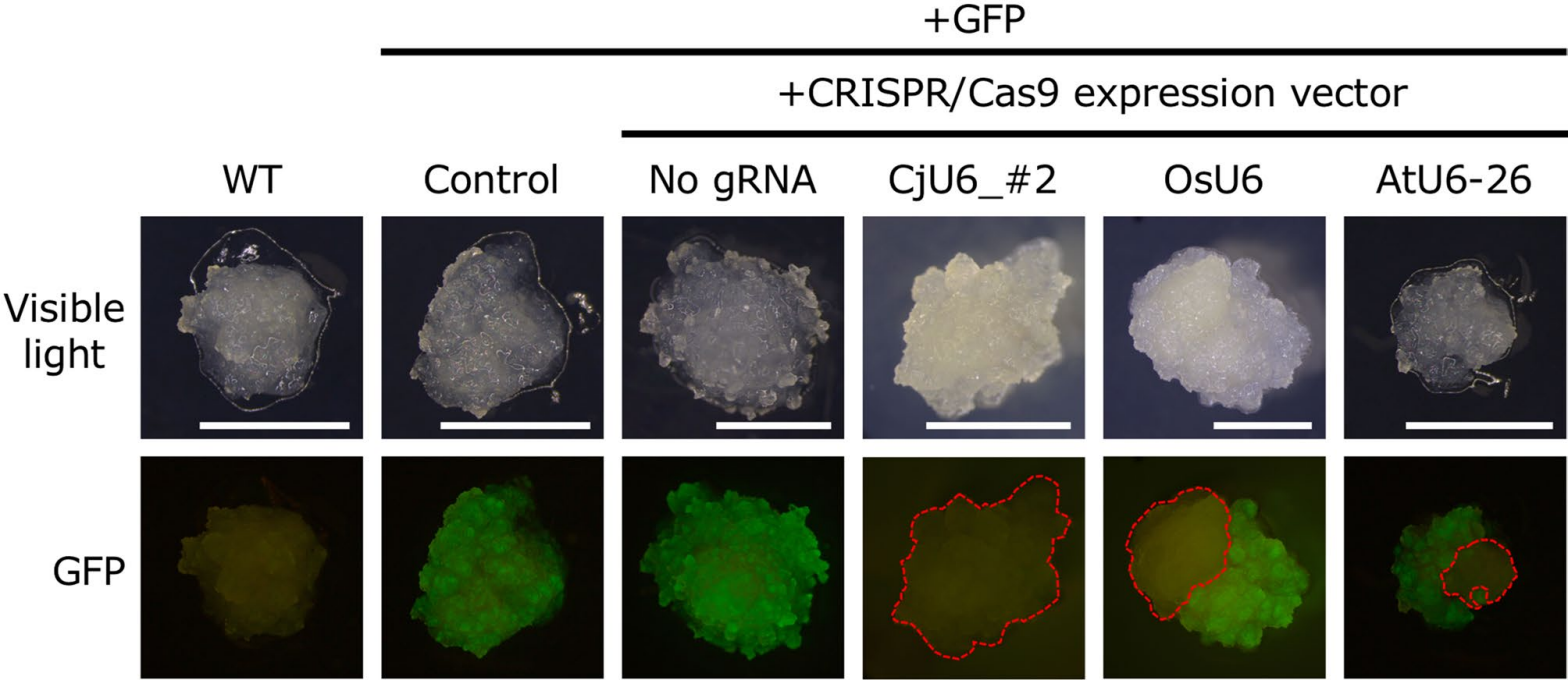
Targeted mutagenesis of GFP in *Cryptomeria japonica* embryogenic tissue (ET). WT, wild-type cell line #13-8-12; + GFP, GFP-overexpressing line N4; + CRISPR/Cas9 expression vector, transgenic N4 lines containing pZF_FFCas9-derived GFP-targeting vectors; No gRNA, ET line #48 containing pZK_FF without the gRNA expression cassette; CjU6_#2, ET line #50 containing pZK_FF with the CjU6_#2-driven GFP target 1 expression cassette vector; OsU6, ET line #27 containing pZK_FF with the OsU6-driven GFP target 1 expression cassette vector; and AtU6-26, ET line #70 containing pZK_FF with the AtU6-26-driven GFP target 1 expression cassette vector. Area enclosed by red dots indicates cells without GFP fluorescence. Bars, 5 mm.

**Figure 3:**
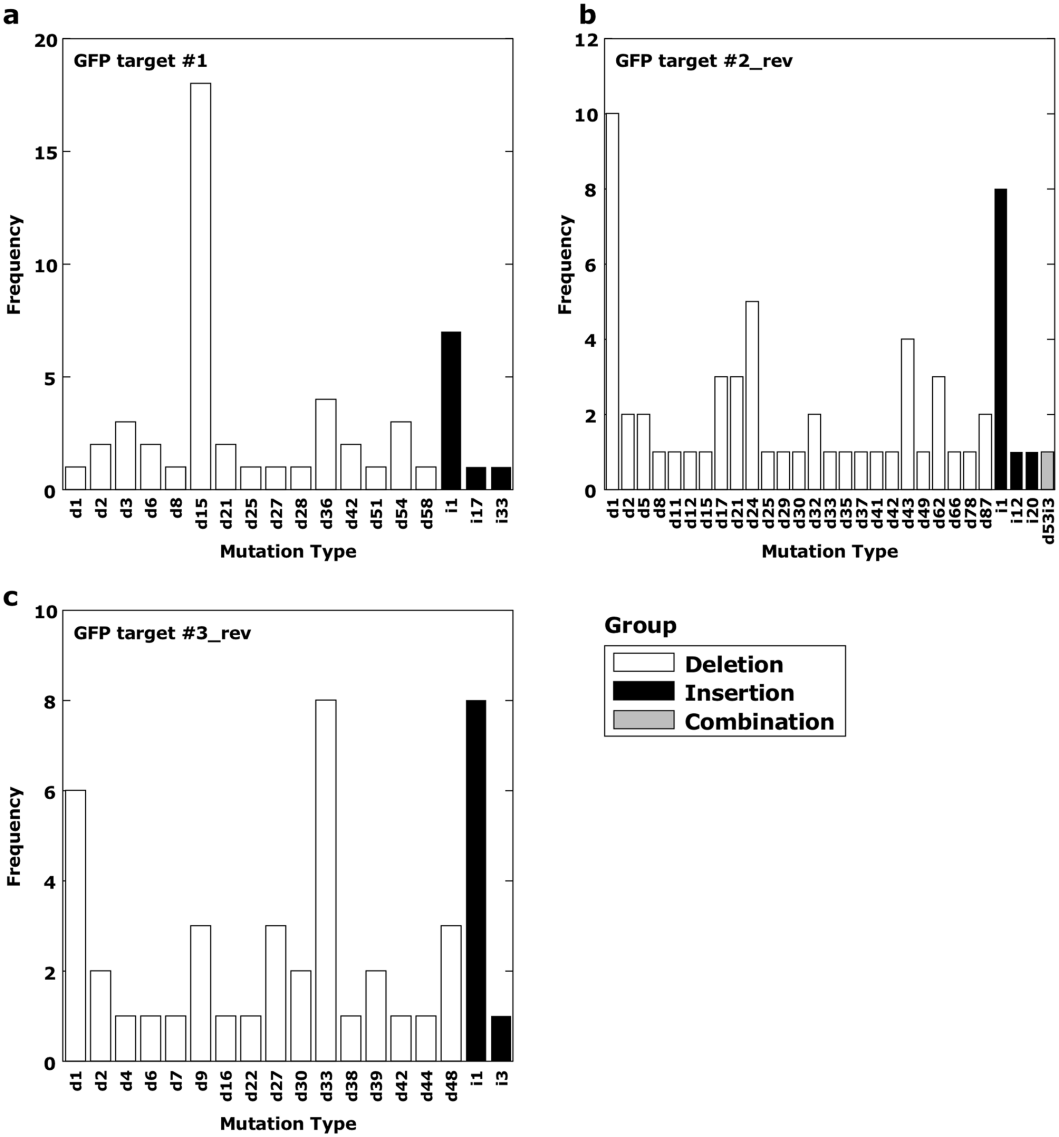
CRISPR/Cas9-induced target-dependent knock-out mutation type and length. pZK_FFCas9 vectors containing the gRNA of GFP target #1 (**a**), #2_rev (**b**) and #3_rev (**c**) driven by AtU6-26 promoters were introduced into the GFP-expressing embryogenic tissue line N4, respectively. d# and i# denote the number of bp deleted and inserted at the target site, respectively. Group: deletion (white bar), mutation pattern containing only deletions; insertion (black bar), mutation pattern containing only insertions; combination (gray bar), mutation pattern containing both deletions and insertions.

To determine the efficiency of the CRISPR/Cas9 system in *C. japonica*, we first obtained an optimal promoter for driving the expression of *SpCas9*. For this, we screened seven constitutive promoters (Supplementary Information) using the dual luciferase (LUC) transient expression assay. PcUbi was found to be the most active (Supplementary Fig. S2) in the ET of *C. japonica* and considered the optimal promoter for driving *SpCas9* expression. Hereafter, PcUbi was used as the promoter for *Cas9* (Fig. 1). Next, to screen for the optimum U6 promoter for gRNA expression, we isolated endogenous U6 promoters in *C. japonica* using TAIL-PCR^35^ with specific primers corresponding to U6 snRNA consensus sequences and isolated 11 putative U6 promoter fragments (Supplementary Information). Sequence analysis of these fragments revealed that all had an upstream sequence element (USE) and TATA-like box sequence, which are typical characteristics of U6 promoters^36^ (Supplementary Fig. S3). To test U6 promoter activity, we used the single-copy GFP-expressing ET line N4 (Supplementary Fig. S1), for the CRISPR/Cas9-mediated GFP knock-out experiment. We selected target sites that have been previously reported^37–39^ (Table 1). pZK_FFCas9 vectors containing the gRNA of GFP target #1 driven by 13 different U6 promoters were introduced into N4, and 17–138 transgenic ET lines were obtained for each vector (Table 2) by screening in a medium with 25 mg/L kanamycin. Loss of GFP fluorescence was observed in 1–39 lines for each independent transgene insertion (Table 2). GFP fluorescence was lost in some lines completely, with others exhibiting GFP fluorescence in some areas of the cell mass (Fig. 2). By contrast, all transgenic lines with the Cas9 expression cassette alone (without the gRNA expression cassette) exhibited GFP fluorescence (Fig. 2, Table 2, Supplementary Fig. S4). GFP knock-out efficiency and number of lines isolated varied within U6 promoters (range 3.1–37.9%) (Table 2). Target-dependent knock-out efficiency using the *Arabidopsis* U6 promoter (Table 3) was 41.4% and 31.2% using target #2_rev and target #3_rev, respectively, which were higher than that for target #1 (25.0%). Mutation patterns in each line were confirmed through sequencing (Fig. 3, Supplementary Fig. S5). Frequent modifications in all targets were single-nucleotide insertions. Additionally, single-nucleotide deletions were often observed in target #2_rev and target #3_rev. By contrast, large deletions, such as 15 bp deletions in target #1 and 33 bp deletion in target #3_rev, were the most frequent modifications. From the point onward, CjU6_#2 promoter was used for gRNA expression in *C. japonica*.

**Table 2:** Effect of U6 promoters on the efficiency of targeted mutagenesis of the GFP transgene.

Target	U6 promoter	No. of transformed lines (A)	No. of GFP knock out lines^∗^ (B)	Efficiency^∗∗^, ^¦^ (%)
#1	–^§^	63	0	0
	CjU6_#1	138	39	28.3^a^
	CjU6_#2	134	33	24.6^a^
	CjU6_#3	98	3	3.1^c^
	CjU6_#4	29	11	37.9^a^
	CjU6_#5	72	15	20.8^a,b^
	CjU6_#6	39	4	10.3^a,b,c^
	CjU6_#7	55	9	16.4^a,b,c^
	CjU6_#8	20	1	5.0^a,b,c^
	CjU6_#9	17	3	17.6^a,b,c^
	CjU6_#10	116	18	15.6^a,b,c^
	CjU6_#11	60	2	3.3^b,c^
	OsU6	50	12	24.0^a,b^
	AtU6-26	136	34	25.0^a^

^∗^The number of calli that completely lost GFP fluorescence or had GFP fluorescence in some parts of the cell mass was counted after 87 days of selective cultivation.

^∗∗﻿^(B/A) × 100.

¦Pairwise multiple comparison of proportions was performed using Tukey’s multiple comparison test. Proportions with significant difference were labeled with different letters (P < 0.05).

§N4 transgenic lines containing the pZK_FFCas9 vector without the gRNA expression cassette (Negative control).

**Table 3:** Effect of target sequence on the efficiency of targeted mutagenesis of the GFP transgene.

Target^∗^	No. of transformed lines (A)	No. of GFP-knock-out lines^∗∗^ (B)	Mutation frequency (%)^¦^	No. of lines having multiple mutation patterns^§^ (C)	Mosaicism frequency (%)^¶^
#1	136^¿^	34^¿^	25.0^¿,b^	15	44.1^c^
#2_rev	70	29	41.4^a^	17	58.6^c^
#3_rev	77	24	31.2^a,b^	15	62.5^c^

*ET* embryogenic tissue.

∗pZK_FFCas9 vectors with gRNA expression cassettes driven by AtU6-26 promoter were transformed into ET.

^∗∗^The number of ET lines was counted after 87 days of selective cultivation.

^¦^(B/A) × 100. Pairwise multiple comparison of proportions was performed using Tukey’s multiple comparison test. Proportions with significant difference were labeled with different letters (P < 0.05).

§The number of ET lines having multiple mutation patterns was counted.

^¶^(C/B) × 100. Pairwise multiple comparison of proportions was performed using Tukey’s multiple comparison test. Proportions with a significant difference were labeled with different letters (P < 0.05).

^¿^Data from Table 1.

### Investigation of chimerism in the regenerated T_0_ plantlet

**Figure 4:**
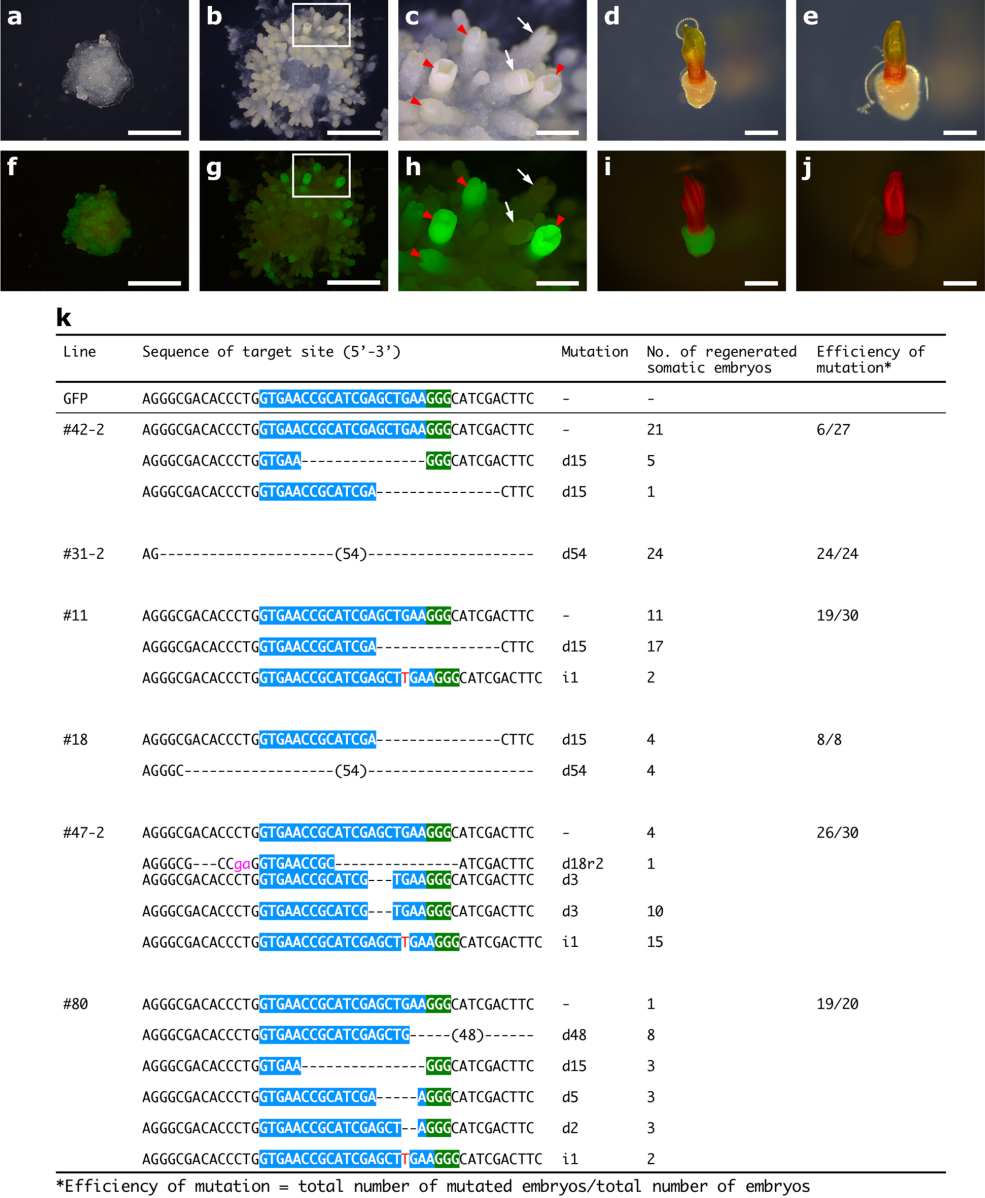
Serial observation of somatic embryogenesis and regeneration from embryogenic tissue (ET) line #42, exhibiting patchy GFP fluorescence under visible light (**a–e**) and blue light (**f–j**), in which pZK_FFCas9 vector containing the gRNA of GFP target #1 driven by CjU6_#2 promoter was introduced. (**a**,**f**) Day 0, ET line #42 in the somatic embryo maturation medium. Bars, 5 mm. (**b**,**g**) Day 39, induction of cotyledonary somatic embryos in the same medium. GFP fluorescence is partially quenched in the cell mass. Bars, 5 mm. (**c**,**h**) Higher magnifications of the framed boxes in (**b)** and (**g**), respectively. White arrows indicate GFP-knock-out embryos. Red arrowheads indicate embryos with active GFP. Bars, 1 mm. (**d**,**e**,**i**,**j**) Germination of cotyledonary somatic embryos at 7 days after culture on germination medium. (**d**,**i**) Embryo with active GFP. Bars, 2 mm. (**e**,**j**) GFP-knock-out embryo. Bars, 2 mm. (**k**) Genotype of regenerated somatic cotyledonary embryos from selected ET lines. The target region and PAM sequence are highlighted in blue and green, respectively. A deletion is denoted by “−”. Letters in red and lower-case pink indicate inserted and replaced bases, respectively. d#, i#, and r# denote the number of bp deleted, inserted, and replaced at the target site, respectively. We extracted genomic DNAs from the whole embryos and determined the nucleotide sequences by direct sequencing.

Loss of GFP fluorescence was frequently partial in many transformed lines (Fig. 2) and ranged from 44.1 to 62.5% depending on the target sequence (Table 3). This is because mutations occur independently in a single cell; therefore, a variety of mutations can be obtained from a single transgenic line^40^. If the plantlet was derived from genomes of edited and non-edited cells, the plantlet may exhibit chimerism or mosaicism, such that the plantlet will have functional GFP in one portion and mutated non-functional GFP in the other portion. During the development of somatic cotyledonary embryos, GFP fluorescence clearly separated into positive or negative; based on our observations, no cotyledon exhibited chimeric GFP fluorescence (Fig. 4a–j). In a green germinating somatic embryo with active GFP, GFP fluorescence could hardly be observed due to strong chlorophyll autofluorescence; conversely, GFP fluorescence was observed in the callus at the base of the embryo (Fig. 4d,i). To investigate mosaicism in cotyledonary somatic embryos, direct sequencing analysis was performed using 8–30 whole cotyledonary embryos (139 from 6 lines). One cotyledonary embryo from line #47-2 had two modification patterns (d18r2 and d3 in Fig. 4k, Supplementary Fig. S6) in one individual somatic embryo. However, in others, mutation patterns were clearly separated in individual cotyledonary embryos (Fig. 4k). Next, the stability of mutation patterns was analyzed in 10 plantlets regenerated through somatic embryogenesis in each of 4 lines, namely #42-2, #18, #31-2, and #11, by direct sequencing of multiple needle and root segments (Supplementary Fig. S7a). All plantlets regenerated from #31-2 and #11 lines had only one mutation pattern in all tissue segments. By contrast, in lines #42-2 and #18, plantlets with additional mutation(s) in root segments were observed (Supplementary Fig. S7b). No apparent mosaicism in the sequence was detected in these tissues by scanning sequence chromatograms.

### Targeted mutagenesis of an endogenous gene using the CRISPR/Cas9 system

**Figure 5:**
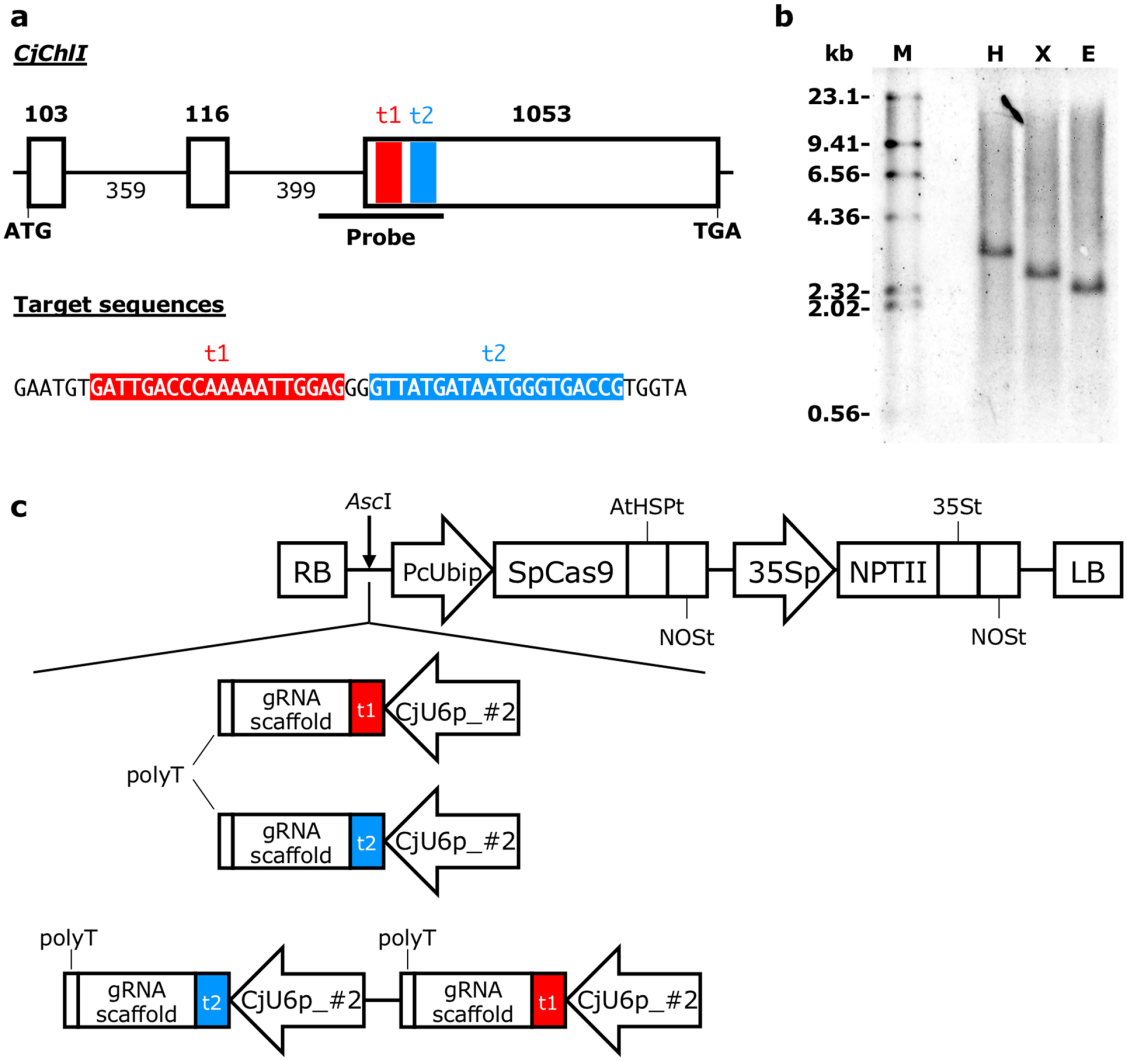
The structure of magnesium chelatase from *Cryptomeria japonica*. (**a**) Genome structure. Red letters show target 1 and blue letters show target 2, respectively. (**b**) Southern blot analysis of endogenous *C. japonica* magnesium chelatase subunit I (*CjChlI*). M; DIG-labeled λ-HindIII marker, H; HindIII, X; XhoI, E; EcoRI. The full-length blot image is included in Supplementary Fig. S8b. (**c**) Schematic representation of pCRG-SpCas9-derived *CjChlI*-targeting vectors.

**Figure 6:**
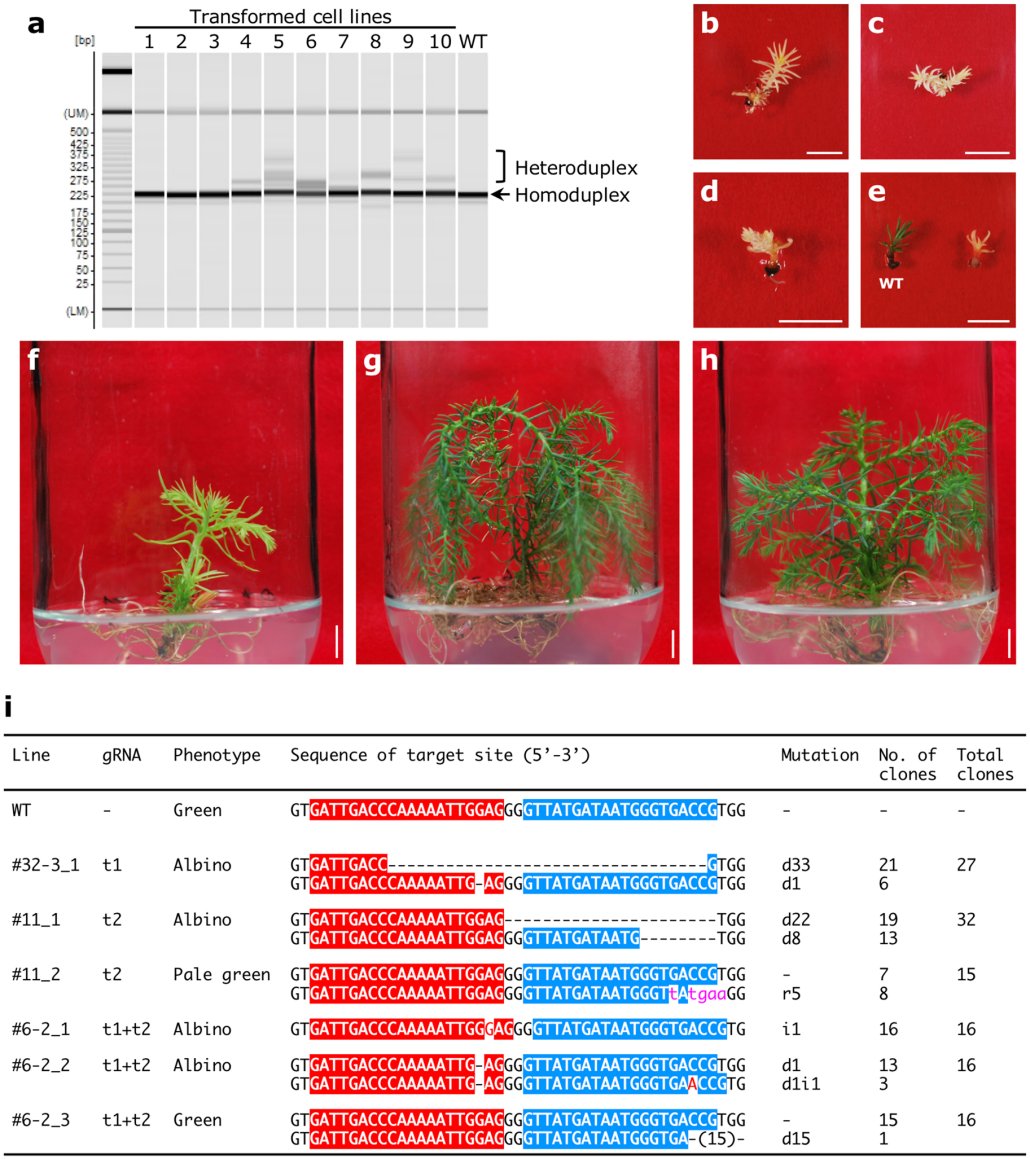
Phenotype associated with CRISPR/Cas9-mediated editing of endogenous *Cryptomeria japonica* magnesium chelatase subunit I (*CjChlI*). (**a**) Heteroduplex mobility assay of 10 transformed embryogenic tissue (ET) lines with pCRG-SpCas9-derived *CjChlI*-targeting vectors and a wild-type ET line #13-8-12 (WT). The formation of DNA heteroduplexes was observed in putative gene-edited lines (#4-10). Regenerated plantlets from putative genome-edited line pCRG-SpCas9-t1_#32-3_1 (**b**), pCRG-SpCas9-t2_#11_1 (**c**), pCRG-SpCas9-t1 + t2_#6-2_1 (**d**), pCRG-SpCas9-t1 + t2_#6-2_2 (**e**), pCRG-SpCas9-t2_#11_2 (**f**), and pCRG-SpCas9-t1 + t2_#6-2_3 (**g**). A regenerated plantlet of wild-type ET line #13–8-12 (**h**). The seedling on the left in (**e**) is WT line #13-8-12. Bars, 1 cm. Plantlets of (**b–d**), and (**f–h**) were grown for 231 days on germination medium, whereas plantlets of (**e**) were on germination medium only for 45 days. (**i**) Mutation pattern in each gene-edited line. Target site sequences t1 and t2 are highlighted in red and blue, respectively. Letters in red and lower-case pink indicate inserted and replaced bases, respectively. d#, i#, and r# denote the number of bp deleted, inserted, and replaced at the target site, respectively. We extracted genomic DNAs from the shoots and amplified a fragment containing the target sequence using primers p#468_f and p#648_r (Supplementary Table S1). The sequence in each clone was analyzed.

We modified an endogenous *C. japonica* gene using CRISPR/Cas9. We selected *ChlI*, which is required for chlorophyll biosynthesis^23^. *ChlI* mutants exhibit an albino phenotype^24^. A 2152 bp fragment corresponding to *CjChlI* was cloned (Accession no. LC603656) (Fig. 5a). Southern blot analysis suggested that *CjChlI* is a single gene (Fig. 5b). To introduce mutations in *CjChlI*, we designed two targeting sequences in exon 3 of *CjChlI*, named t1 and t2, respectively (Fig. 5a). Three binary vectors for disrupting *CjChlI* were constructed using the pCRG-SpCas9 vector (Fig. 5c, Supplementary Information). Transgenic lines were selected in a kanamycin-containing medium. Then, putative genome-edited lines were screened using the heteroduplex mobility assay (HMA) (Fig. 6a), leading to the selection of 7, 4, and 2 lines for t1, t2, and t1 + t2, respectively (Table 4). Transgenic plantlets were induced through mature somatic embryos; some lines failed to produce somatic embryos, and regenerated shoots with visible phenotypes, namely albino, pale green, and green (Fig. 6b–g). Albino transformants (Fig. 6b–e) grew very slowly. Pale green transformant (Fig. 6f) grew slower than wild-type. A green transformant (Fig. 6g) grew similar to wild-type (Fig. 6h). To verify the modification of sequences at target sites in transformants induced by the CRISPR/Cas9 system, sequences around the target site were analyzed from genomic DNA isolated from aerial part of transformants. PCR products were inserted into a cloning vector and 15–32 clones were analyzed for each transformant. Various mutations were detected at target sites in these lines (Fig. 6i), indicating that targeted mutagenesis of *CjChlI* occurred. Moreover, *CjChlI* was completely disrupted in albino transformants. By contrast, a point mutation was observed in 8 of 15 clones in the pale green transformant. In the green transformant (t1 + t2_#6-2_3), 1 of 16 clones had a deletion in the target site. Taken together, our results indicate that the CRISPR/Cas9 system induced targeted mutagenesis through double-strand breaks in an endogenous gene of *C. japonica*.

**Table 4:** Summary of endogenous *Cryptomeria japonica* magnesium chelatase subunit I (*CjChlI*) modification using the CRISPR/Cas9 system.

Target^∗^	No. of transformed lines (A)	No. of HMA positive lines (B)	Mutation frequency (%)^∗∗^
t1	20	7	35^a^
t2	17	4	23.5^a^
t1 + t2	7	2	28.6^a^

^∗^pCRG-SpCas9 vectors with gRNA expression cassettes driven by CjU6_#2 promoter were transformed into embryogenic tissue.

^∗∗^(B/A) × 100. Pairwise multiple comparison of proportions was performed using Tukey’s multiple comparison test. Proportions with significant difference were labeled with different letters (P < 0.05).

## Discussion

Here we report the first example of targeted mutagenesis through the CRISPR/Cas9 system in *C. japonica*. This is also the first reported case of a gene-edited coniferous trees species to the best of our knowledge. Genetic transformation of conifers is expected to be a powerful tool for modifying their characteristics in a short time. This technology has been used for more than two decades to produce herbicide-tolerant and insect-resistant crops, such as corn, soybean, and cotton^41^. Although examples of genetically modified trees have accumulated^42^, there are not so much field trial researches on long-term behavior (more than 10 years) of transgenes in genetically modified trees. In addition, unlike crop cultivation, trees grow in forests, where human management is a challenge. Studies have also raised concerns about transgene instability^43^. Genome-edited plants with no transgenes are attractive for perennial tree species in terms of public acceptance. Inducing gibberellin-mediated flowering in *C. japonica* is easy. Among conifers, obtaining null segregants through mating in a relatively short time is possible in *C. japonica*, because it is easy to induce earlier flowering in even 1-year-old seedling plants by gibberellin treatment^44^. Moreover, *C. japonica*, which has a stable culture, transformation system, and genome editing system, could be a model for coniferous tree species.

During vector development, AtHSP and NOS terminators in tandem were found to be important for the stable expression of exogenous proteins. The pZmUbi-GFP-Dt vector-introducing lines maintained strong GFP fluorescence after 5 years of subculture. In this study, we also surveyed the activity of U6 promoters isolated from *C. japonica*, in addition to the commonly used ones from *A. thaliana* and *Oryza sativa*, for improving mutation efficiency. In some plant species, such as soybean and cotton, mutation efficiency increased when their intrinsic U6 promoters were used instead of the *Arabidopsis* U6 promoter^45,46^. However, contrary to expectations, the efficiency of genome editing using the U6 promoters of *C. japonica* was not significantly different from that with the U6 promoters of *A. thaliana* or *O. sativa* (Table 2). Whether this was due to the activity of the U6 promoter or the expression level of Cas9 is unknown. The terminator used for the Cas9 expression cassette in pZK_FFCas9 (Fig. 1b) might be unsuitable for Cas9 expression, compared with the tandem terminator unit of AtHSPt and NOSt in the pCRG-SpCas9 vector (Fig. 5c). Whether our results apply to all conifers warrants further study using other target tree species.

In the GFP-expressing line (N4), mutation frequency with GFP target #2_rev was 41.4% (Table 3). By contrast, Jacob et al.^39^ detected indels in more than 70% transgenic lines when targeting the same GFP region in a genome editing study using GFP-expressing hairy roots of soybeans. These results may indicate that the amount of Cas9 in *C. japonica* was lower than that in soybean. Short indel patterns were observed in a large number of transgenic lines (Fig. 3, Supplementary Fig. S5), which is consistent with the results of others^21,47^. In addition, some transgenic lines had large deletions, such as 15 and 33 bp in GFP target #1 and target #3_rev, respectively (Fig. 3 and Supplementary Fig. S5). This deletion may be a microhomology-associated deletion^48^, that is, microhomologies, such as “TCGA” and “GCTG,” between the target sequence in GFP (Supplementary Fig. S5). Chimerism or mosaicism is an important concern in the application of genome editing in plant species, especially when generation intervals are counted in years. Transgenic lines with mottled GFP knock-out regions were used to investigate chimerism in induced somatic embryos; however, 101 of 102 mutated embryos, more than 99% of embryos had a single modification pattern (Fig. 4k). The results support the hypothesis that the somatic embryo culture system originated from a single cell^49^. However, we also observed new modifications in the root tissue as the individual grew larger (Supplementary Fig. S7b). This result suggests that Cas9-mediated DNA cleavage is ongoing. In line #18, two modification patterns (d15 and d54) were observed in cotyledonary embryos; however, the d54 mutation pattern was not observed in plantlets (Fig. 4k and Supplementary Fig. S7). One possible reason for this result is that the cell masses with the d54 mutation were discarded in the process of serial subculture. To maintain cell lines containing multiple mutation patterns, it might be necessary to pick cell masses from several locations during subculture. Furthermore, the promoter activity of PcUbi is expected to be higher in the root tissue than in ET and needle tissue. These results also imply that if mutated embryos could be isolated, the chimerism would be eliminated. A difference in editing efficiency depending on the target sequence was observed (Table 3). Doench et al.^50^ developed a tool to predict gRNA activity; however, no correlation between the score value and actual cutting efficiency was observed. No effective tool is available for predicting gRNA cleavage efficiency in plants^51^.

We succeeded in performing targeted mutagenesis of endogenous *CjChlI* (Fig. 6b–g). In addition, a mutation was observed in one of 16 clones from the green transformant line t1 + t2_#6-2_3 (Fig. 6g,i). This result indicates that continuous DNA cleavage by Cas9 and prolonged cultivation can lead to the modification of target regions. Further improvements in the CRISPR/Cas9 expression vector are required for more efficient mutagenesis. It has previously been reported that attaching multiple Cas9s near the target sequence increased the editing efficiency of the target sequence due to the helicase activity of Cas9^52^. Based on this result, we predicted that genome editing efficiency would be improved when gRNAs were designed to be adjacent to the target region and expressed simultaneously. However, since the number of regenerated individuals was small, we are unable to evaluate our hypothesis at this time. Codon optimization of Cas9 dramatically increases the efficiency of obtaining transformants with mutations in both alleles (Nanasato et al., in preparation). We are conducting genome editing studies using the improved vector and producing genome-edited male-sterile lines. The use of genome editing technology will directly contribute to the analysis of gene function in conifers, which has been mostly unfeasible until now.

To achieve the full potential of genome editing technologies in conifer species, detailed genomic information needs to be available. In addition, an efficient technique for producing transgene-free individuals, through methods other than crossing, must be developed for practical use. In many crops, crossing has been used to obtain transgene-free individuals. However, crossing in conifers takes years. Furthermore, conifer species are not autogamous, and during crossing with another individual, useful parental traits are lost in the offspring. Direct delivery of the Cas9 and sgRNA complex into plant cells is an attractive solution^53,54^. The cell wall is a major obstacle to the direct delivery of proteins. Yanagawa et al.^55^ used a multi-gas plasma jet to deliver proteins into tobacco leaf. We have discovered a cell-penetrating peptide, which delivers proteins directly into *C. japonica* cells^56^. We believe that the integration of the efficient genome editing system we report here and direct protein delivery techniques will accelerate basic research and molecular breeding in conifers.

## Supplementary Information

Supplementary Information.

## References

[1] Tsumura Y (2020). Effects of the last glacial period on genetic diversity and genetic differentiation in *Cryptomeria japonica* in East Asia. Tree Genet. Genomes.

[2] Ohba, K. Clonal forestry with sugi (*Cryptomeria japonica*). In *Clonal Forestry II* (eds. Ahuja, M. & Libby, W.) 66–90 (Springer, 1993). 10.1007/978-3-642-84813-1_4

[3] Kondo T, Kuramoto N, Kole C (2007). *Cryptomeria japonica*. Forest Trees.

[4] Forest Agency, Japan. *State of Japan’s Forests and Forest Management—3rd Country Report of Japan to the Montreal Process*. http://www.rinya.maff.go.jp/j/kaigai/pdf/countryreport-1.pdf. Accessed 30 Jul 2021 (2019).

[5] Yamada T, Saito H, Fujieda S (2014). Present state of Japanese cedar pollinosis: The national affliction. J. Allergy Clin. Immunol..

[6] Tsubomura M, Kurita M, Watanabe A (2016). Determination of male strobilus developmental stages by cytological and gene expression analyses in Japanese cedar (*Cryptomeria japonica*). Tree Physiol..

[7] Futamura, N., Igasaki, T., Saito, M., Taira, H. & Shinohara, K. Comparison of fertile and sterile male gametogenesis in *Cryptomeria japonica* D. Don. *Tree Genet. Genomes* **15**, 30 (2019).

[8] Hasegawa Y (2018). Fine mapping of the male-sterile genes (MS1, MS2, MS3, and MS4) and development of SNP markers for marker-assisted selection in Japanese cedar (*Cryptomeria japonica* D. Don). PLoS One.

[9] Hasegawa Y (2021). Identification and genetic diversity analysis of a male-sterile gene (MS1) in Japanese cedar (*Cryptomeria japonica* D. Don). Sci. Rep..

[10] Mishima K (2018). Identification of novel putative causative genes and genetic marker for male sterility in Japanese cedar (*Cryptomeria japonica* D. Don). BMC Genom..

[11] Maruyama TE, Ueno S, Hirayama S, Kaneeda T, Moriguchi Y (2020). Somatic embryogenesis and plant regeneration from sugi (Japanese cedar, *Cryptomeria japonica* D. Don, Cupressaceae) seed families by marker assisted selection for the male sterility allele *ms1*. Plants.

[12] Jinek M (2012). A programmable dual-RNA—Guided DNA endonuclease in adaptive bacterial immunity. Science.

[13] Shan S, Soltis PS, Soltis DE, Yang B (2020). Considerations in adapting CRISPR/Cas9 in nongenetic model plant systems. Appl. Plant Sci..

[14] Nishimasu H (2018). Engineered CRISPR-Cas9 nuclease with expanded targeting space. Science.

[15] Walton RT, Christie KA, Whittaker MN, Kleinstiver BP (2020). Unconstrained genome targeting with near-PAMless engineered CRISPR-Cas9 variants. Science.

[16] Ran FA (2015). In vivo genome editing using *Staphylococcus aureus* Cas9. Nature.

[17] Pausch P (2020). CRISPR-CasΦ from huge phages is a hypercompact genome editor. Science.

[18] Nishida K (2016). Targeted nucleotide editing using hybrid prokaryotic and vertebrate adaptive immune systems. Science.

[19] Gaudelli NM (2017). Programmable base editing of A•T to G•C in genomic DNA without DNA cleavage. Nature.

[20] Anzalone AV (2019). Search-and-replace genome editing without double-strand breaks or donor DNA. Nature.

[21] Fan D (2015). Efficient CRISPR/Cas9-mediated targeted mutagenesis in *Populus* in the first generation. Sci. Rep..

[22] Zhou X, Jacobs TB, Xue L-J, Harding SA, Tsai C-J (2015). Exploiting SNPs for biallelic CRISPR mutations in the outcrossing woody perennial *Populus* reveals 4-coumarate: CoA ligase specificity and redundancy. New Phytol..

[23] Kruse E, Mock H, Grimm B (1997). Isolation and characterisation of tobacco (*Nicotiana tabacum*) cDNA clones encoding proteins involved in magnesium chelation into protoporphyrin IX. Plant Mol. Biol..

[24] Koncz C (1990). Isolation of a gene encoding a novel chloroplast protein by T-DNA tagging in *Arabidopsis thaliana*. EMBO J..

[25] Taniguchi T, Ohmiya Y, Kurita M, Tsubomura M, Kondo T (2008). Regeneration of transgenic *Cryptomeria japonica* D. Don after *Agrobacterium tumefaciens*-mediated transformation of embryogenic tissue. Plant Cell Rep..

[26] Konagaya KI, Nanasato Y, Taniguchi T (2020). A protocol for Agrobacterium-mediated transformation of Japanese Cedar, Sugi (*Cryptomeria japonica* D. Don) using embryogenic tissue explants. Plant Biotechnol..

[27] Nagaya S, Kawamura K, Shinmyo A, Kato K (2010). The HSP terminator of *Arabidopsis thaliana* increases gene expression in plant cells. Plant Cell Physiol..

[28] Mikami M, Toki S, Endo M (2015). Parameters affecting frequency of CRISPR/Cas9 mediated targeted mutagenesis in rice. Plant Cell Rep..

[29] Fauser F, Schiml S, Puchta H (2014). Both CRISPR/Cas-based nucleases and nickases can be used efficiently for genome engineering in *Arabidopsis thaliana*. Plant J..

[30] Kuroda M, Kimizu M, Mikami C (2010). A simple set of plasmids for the production of transgenic plants. Biosci. Biotechnol. Biochem..

[31] Endo M, Nishizawa-Yokoi A, Toki S, Murata M (2016). Targeted mutagenesis in rice using TALENs and the CRISPR/Cas9 system. Chromosome and Genomic Engineering in Plants: Methods and Protocols.

[32] Kawalleck P, Somssich IE, Feldbrügge M, Hahlbrock K, Weisshaar B (1993). Polyubiquitin gene expression and structural properties of the *ubi*4-2 gene in *Petroselinum crispum*. Plant Mol. Biol..

[33] Hyun Y (2014). Site-directed mutagenesis in *Arabidopsis thaliana* using dividing tissue-targeted RGEN of the CRISPR/Cas system to generate heritable null alleles. Planta.

[34] Konagaya KI, Kurita M, Taniguchi T (2013). High-efficiency Agrobacterium-mediated transformation of *Cryptomeria japonica* D. Don by co-cultivation on filter paper wicks followed by meropenem treatment to eliminate Agrobacterium. Plant Biotechnol..

[35] Liu Y-G, Mitsukawa N, Oosumi T, Whittier RF (1995). Efficient isolation and mapping of *Arabidopsis thaliana* T-DNA insert junctions by thermal asymmetric interlaced PCR. Plant J..

[36] Waibel F, Filipowicz W (1990). U6 snRNA genes of *Arabidopsis* are transcribed by RNA polymerase III but contain the same two upstream promoter elements as RNA polymerase II-transcribed U-snRNA genes. Nucleic Acids Res..

[37] Fu Y (2013). High-frequency off-target mutagenesis induced by CRISPR-Cas nucleases in human cells. Nat. Biotechnol..

[38] Mali P (2013). RNA-guided human genome engineering via Cas9. Science.

[39] Jacobs TB, Lafayette PR, Schmitz RJ, Parrott WA (2015). Targeted genome modifications in soybean with CRISPR/Cas9. BMC Biotechnol..

[40] Sugano SS (2017). Genome editing in the mushroom-forming basidiomycete *Coprinopsis cinerea*, optimized by a high-throughput transformation system. Sci. Rep..

[41] Kumar K (2020). Genetically modified crops: Current status and future prospects. Planta.

[42] Strauss SH (2017). Reproductive modification in forest plantations: Impacts on biodiversity and society. New Phytol..

[43] Hoenicka, H. & Fladung, M. Genome instability in woody plants derived from genetic engineering. In *Tree Transgenesis* (eds. Fladung, M. & Ewald, D.) 301–321 (Springer, 2006). 10.1007/3-540-32199-3_14.

[44] Hashizume H (1959). The effect of gibberellin upon flower formation in *Cryptomeria japonica*. J. Jpn. For. Soc..

[45] Long L (2018). Optimization of CRISPR/Cas9 genome editing in cotton by improved sgRNA expression. Plant Methods.

[46] Sun X (2015). Targeted mutagenesis in soybean using the CRISPR-Cas9 system. Sci. Rep..

[47] Feng Z (2014). Multigeneration analysis reveals the inheritance, specificity, and patterns of CRISPR/Cas-induced gene modifications in *Arabidopsis*. Proc. Natl. Acad. Sci. U. S. A..

[48] Bae S, Kweon J, Kim HS, Kim JS (2014). Microhomology-based choice of Cas9 nuclease target sites. Nat. Methods.

[49] Nagmani R, Becwar MR, Wann SR (1987). Single-cell origin and development of somatic embryos in *Picea abies* (L.) Karst. (Norway spruce) and *P. glauca* (Moench) Voss (white spruce). Plant Cell Rep..

[50] Doench JG (2014). Rational design of highly active sgRNAs for CRISPR-Cas9-mediated gene inactivation. Nat. Biotechnol..

[51] Naim F (2020). Are the current gRNA ranking prediction algorithms useful for genome editing in plants?. PLoS One.

[52] Chen F (2017). Targeted activation of diverse CRISPR-Cas systems for mammalian genome editing via proximal CRISPR targeting. Nat. Commun..

[53] Woo JW (2015). DNA-free genome editing in plants with preassembled CRISPR-Cas9 ribonucleoproteins. Nat. Biotechnol..

[54] Svitashev S, Schwartz C, Lenderts B, Young JK, Mark Cigan A (2016). Genome editing in maize directed by CRISPR–Cas9 ribonucleoprotein complexes. Nat. Commun..

[55] Yanagawa Y (2017). Direct protein introduction into plant cells using a multi-gas plasma jet. PLoS One.

[56] Tanaka Y (2021). Direct protein delivery into intact plant cells using polyhistidine peptides. Biosci. Biotechnol. Biochem..

