# Supplementary material for: CRISPR/Cas9-mediated targeted mutagenesis in Japanese cedar (Cryptomeria japonica D. Don)
Source: Sci Rep. 2021 Aug 10;11:16186. doi: 10.1038/s41598-021-95547-w (PMC8355236; doi:10.1038/s41598-021-95547-w)
Supplement: Supplementary file 1 — Supplementary Information. [file 41598_2021_95547_MOESM1_ESM.pdf]

Supplementary Information

CRISPR/Cas9-mediated targeted mutagenesis in Japanese cedar (*Cryptomeria japonica* D. Don)

Yoshihiko Nanasato[1*], Masafumi Mikami[2,3], Norihiro Futamura[4], Masaki Endo[2,3,5], Mitsuru Nishiguchi[4], Yasunori Ohmiya[4], Ken-ichi Konagaya[1], Toru Taniguchi[1,†]

[1]Forest Bio Research Center, Forestry and Forest Products Research Institute, 3809-1 Ishi, Juo, Hitachi, Ibaraki 319-1301, Japan
[2]Graduate School of Nanobioscience, Yokohama City University, 22-2 Seto, Yokohama, Kanagawa 236-0027, Japan

[3]Plant Genome Engineering Research Unit, Institute of Agrobiological Sciences, National Agriculture and Food Research Organization, 1-2 Owashi, Tsukuba, Ibaraki 305-8634, Japan

[4]Department of Forest Molecular Genetics and Biotechnology, Forestry and Forest Products Research Institute, 1 Matsunosato, Tsukuba, Ibaraki 305-8687, Japan

[5]Kihara Institute for Biological Research, Yokohama City University, 641-12 Maioka-cho, Yokohama, Kanagawa 244-0813, Japan

[*]Correspondence and requests for materials should be addressed to Y.N. (e-mail: nanasato@affrc.go.jp)

[†]Present address: Tohoku Regional Office, Forest Tree Breeding Center, Forestry and Forest Products Research Institute, 95 Osaki, Takizawa, Iwate 020-0621, Japan

Supplementary Information contains:

Supplementary Figures S1-S8, Supplementary Tables S1 and S2, CjU6 promoter and snRNA sequences, CjEF1α promoter sequence

Transient expression analysis for quantification of promoter activity in *C. japonica*

Promoter activity was determined with a transient luciferase reporter assay. The vector 35Sp::LUC-HSPt, containing the firefly luciferase gene (LUC) under the control of the CaMV35S promoter and a heat shock protein terminator from *Arabidopsis thaliana*[1] (HSPt), was derived from 35S::LUC[2]. The vector 35Sp::RLUC-HSPt, containing the *Renilla reniformis* luciferase gene (RLUC) under the control of the CaMV35S promoter and HSPt, was derived from 35S::RUC[2]. The promoter fragment of elongation factor 1 alpha (*CjEF1α*) was isolated from *C. japonica* through thermal asymmetric interlaced polymerase chain reaction[3] (TAIL-PCR) with arbitrary common degenerate primers[4], (Supplementary Table S1) using KOD Plus Neo (Toyobo, Osaka, Japan). For the first round of TAIL-PCR, p#7_TAIL1_r_1st was used for the primary reaction, p#8_TAIL1_r_2nd for the secondary reaction, and p#9_TAIL1_r_3rd for the tertiary reaction (Supplementary Table S1). Another two rounds of TAIL-PCR were performed to obtain a longer sequence. For the second round, p#43_TAIL2_r_1st, p#44_TAIL2_r_2nd, and p#45_TAIL2_r_3rd were used for the primary, secondary, and tertiary reactions, respectively. For the third round, p#100_TAIL3_r_1st, p#101_TAIL3_r_2nd, and p#102_TAIL3_r_3rd were used for the primary, secondary, and tertiary reactions, respectively. The resultant 3 kb of 5' flanking sequence of *CjEF1α* was isolated. Selected promoters (2×CaMV35S, El2Ω[5], Nos, ZmUbi[6], PcUbi[7], and CjEF1α) were used to replace the CaMV35S promoter in 35Sp::LUC-HSPt vector, to yield 2×35Sp::LUC-HSPt, El2Ωp::LUC-HSPt, NOSp::LUC-HSPt, ZmUbip::LUC-HSPt, PcUbip::LUC-HSPt, and CjEF1αp::LUC-HSPt, respectively. About 0.25 g fresh weight of embryogenic tissues was suspended in 1/2MD medium[8] and evenly distributed over the surface of a sterile filter paper (7-cm in diameter, No. 7, Advantech, Japan, cat. no. 01701070) with the aid of a Büchner funnel. The filter paper, with tissues. was placed on a Petri dish containing 1/2 MD medium with 4 g/L gelrite. The plasmids and 35Sp::RLUC-HSPt were introduced simultaneously using a particle bombardment system

(Bio-Rad, Hercules, California). After overnight culture at 25 °C under dark, luciferase activity in the tissue was determined using a Dual Luciferase Reporter Assay System (Promega, Madison, Wisconsin) with the GloMax 20/20 Luminometer (Promega). Firefly luciferase values were normalized to the internal transfection control provided by the RLUC activity of 35Sp::RLUC-HSPt.

Statistical analysis

One-way analysis of variance (ANOVA) was adopted for the data analysis. Means were compared by Tukey's honest significant difference (HSD) test to identify significant differences ($P < 0.05$) between the samples.

Isolation of U6 promoters from *C. japonica*

For the amplification of 5' flanking regions of U6 snRNA genes from *C. japonica*, a round of TAIL-PCR was performed with three arbitrary common degenerate primers, namely AD1–AD3[4], and two U6 snRNA gene-specific reverse primers, namely p#23_TAIL_r_1st (for the primary reaction) and p#24_TAIL_r_2nd (for the secondary reaction), designed based on the sequence of U6 snRNA. After sequence analysis of amplified fragments, to confirm whether the isolated fragments contained the U6 promoter, another round of PCR was performed to amplify U6 promoter and U6 snRNA regions from genomic DNA using specific primers. One of the primers was designed based on the 5' region of amplified fragments (p#67-#71) and the other on 3' regions of U6 snRNA genes (p#72_deg_r). PCR conditions were as follows: 94 °C for 2 min; 30 cycles at 98 °C for 10 s, 60 °C for 30 s, and 68 °C for 30 s; and final extension at 68 °C for 7 min. These fragments were cloned using the Zero-blunt PCR II TOPO cloning kit (Invitrogen, Waltham, Massachusetts) and analyzed the sequences. Eleven fragments with U6 snRNA genes were considered U6 promoters (Supplementary Figure S2). To replace these CjU6

promoters with the OsU6 promoter in pUC6gRNA, two rounds of PCR were performed to add additional nucleotides (Supplementary Table 2), and the fragment was inserted in *Spe*I and *Bbs*I sites of pUC6gRNA using In-Fusion cloning (In-Fusion HD Cloning Kit, Takara Bio Inc., Shiga, Japan) to yield pCjU6_#1gRNA–pCjU6_#11gRNA. PCR conditions were the same as above.

Vector construction for *CjChlI* knock-out vectors

The CRISPR/Cas9 target sites in the *CjChlI* gene were screened and selected using the "sgRNA Analysis" tool in the ApE program (https://jorgensen.biology.utah.edu/wayned/ape/). The tool uses a predictive model reported by Doench et al[9]. To generate the gRNA expression cassette, overlapping PCR was performed. pCjU6_#2 was used as template DNA. For target 1 expression cassette, we performed PCR with the primer pair p#353_f and p#333_r and another round of PCR with the primer pair #354_r and p#343_f. PCR conditions were as follows: 94 °C for 2 min; 30 cycles at 98 °C for 10 s, 50 °C for 30 s, and 68 °C for 20 s; and final extension at 68 °C for 7 min. Fragments of approximately 200 bp were purified using the MinElute Gel Extraction Kit (QIAGEN, Venlo, Netherlands) and inserted using In-Fusion cloning with the *Asc*I-cut pCRG-SpCas9 vector to yield pCRG-SpCas9-ChlI-t1. For target 2 expression cassette, two primer pairs, including p#355_f and p#333_r and p#356_r and p#343_f, were used to amplify fragments. PCR cycle used was the same as above. The In-Fusion reaction was performed using the *Asc*I-cut pCRG-SpCas9 vector to obtain pCRG-SpCas9-ChlI-t2. For the tandem insertion of target 1 and target 2 expression cassettes, four fragments were amplified with four primer pairs as follows: #354_r and p#343_f, p#353_f and p#329_T3_r, p#355_f and p#333_r, and p#356_r and p#347_T3_f. PCR cycle used was the same as above. These purified fragments were inserted into pCRG-SpCas9 using the In-Fusion reaction to obtain pCRG-SpCas9-ChlI-t1 + t2.

Supplementary Table S1. List of oligonucleotides used in this study

Name	Sequence (5'-3')	Application
AD1	NGTCGA(G/C)(A/T)GANA(A/T)GAA	Arbitrary degenerate primers for TAIL-PCR
AD2	GTNCGA(G/C)(A/T)CANA(A/T)GTT	
AD3	(A/T)GTGNAG(A/T)ANCANAGA	
p#23_TAIL_r_1st	AAAATTTGGACCATTTCTCGATTTG	Cloning of CjU6 promoters
p#24_TAIL_r_2nd	ATTTGTGCGTGTCATCCTTGCGCAG	
p#67_f	GGTCGAGAGATATGAAGAGCACCCCA	
p#68_f	GTGCGAGTCATATGTTGAGGTGAGG	
p#69_f	CATTGTGGTCGTCGATATGTAGGATG	
p#70_f	AGTGGAGTAGCAGAGACAGAGACAGGA	
p#71_f	GTGCGAGACATATGTTGCAGATAAAATGTT	
p#72_deg_r	AAAATTTGGACCATTTCTCGATTTRT	
p#73_f	GCTCGGATCCACTAGTGGTCGAGAGATATGAAGAG	
p#75_r	CTTCTCGAAGACCCCAAGAAGATGTGTACAAAACA	
p#76_f	GCTCGGATCCACTAGTGTGCGAGTCATATGTTGAG	
p#77_r	CTTCTCGAAGACCCCACAACAGCAGGCGTACAAAA	
p#78_f	GCTCGGATCCACTAGTCATTGTGGTCGTCGATATG	
p#79_r	CTTCTCGAAGACCCCACTTAAATACGTGTACAAAA	
p#107_r	CTTCTCGAAGACCCCACTAGTGGGAGCGTACAAAA	
p#108_r	CTTCTCGAAGACCCCAAGAAGATGTGTACAAAACA	
p#109_r	CTTCTCGAAGACCCCACAACAAGAGGCGTACGAAA	
p#110_r	CTTCTCGAAGACCCCATTAATTTACGCGTACACAA	
p#111_r	CTTCTCGAAGACCCCTACCATAATCGTAAACAGTA	
p#112_r	CTTCTCGAAGACCCCATTCAACTGTCAATACAAAA	
p#96_f	GCTCGGATCCACTAGTAGTGGAGTAGCAGAGACAG	
p#97_r	CTTCTCGAAGACCCCATTGACAATTTGTACAAAAC	
p#98_f	GCTCGGATCCACTAGTGTGCGAGACATATGTTGCA	
p#99_r	CTTCTCGAAGACCCCATTTCAAGGTCACTAGCTCT	
p#74_r	TTCTAGCTCTAAAACAGGTCTTCTCGAAGACCCC	
p#7_TAIL1_r_1st	CTCTTGTTCATCTCAGCAGCTTCCTTCTC	Cloning of CjEF1a promoter
p#8_TAIL1_r_2nd	TAGTAGTTGACTTTCCAGAGTCGACATGAC	
p#9_TAIL1_r_3rd	GAGTCGACATGACCAATGACCACAATGT	
p#43_TAIL2_r_1st	TCAAGCACCCAAGCATACTTGAATGAC	

p#44_TAIL2_r_2nd	CCACGTTCACGCTCAGCCTTTAGCTTA	
p#45_TAIL2_r_3rd	TTTCCCATGGTTTCTTGTAGTCAAAA	
p#100_TAIL3_r_1st	ATATAATAGGAGCAGCAGAGAGGAC	
p#101_TAIL3_r_2nd	AGGTGGAAGAAACCAAGTCACTC	
p#102_TAIL3_r_3rd	GAAGAAAACAAGTAAGGTTCTCTTTG	
p#84_f	GTTGGTGAACCGCATCGAGCTGAA	
p#85_r	AAACTTCAGCTCGATGCGGTTCAC	
p#86_r	GTTGGCTGAAGCACTGCACGCCGT	GFP knock-out vectors
p#87_r	AAACACGGCGTGCAGTGCTTCAGC	
p#88_f	GTTGGGGCACGGGCAGCTTGCCGG	Detection of mutation
p#89_r	AAACCCGGCAAGCTGCCCGTGCCC	in GFP
p#113_f	ATGGCATATGCAGCAGCTATATGT	
p#49_r	GAGCTCCTTATCTTTAATCATATTCCAT	
p#353_f	GATTGACCCAAAAATTGGAGGTTTTAGAGCTAGAA	
p#354_r	CTCCAATTTTTGGGTCAATCCACAACAGCAGGCGT	
p#355_f	GTTATGATAATGGGTGACCGGTTTTAGAGCTAGAA	
p#356_r	CGGTCACCCATTATCATAACCACAACAGCAGGCGT	*ChChlI* knock-out
p#333_r	TACGAATTGGGCGCGGTTGGGTAACGCCAGGGTTT	vectors
p#343_f	TTTAAGCTTGGCGCGCCTTAATTAAGGTACCGAGCTC	
p#329_T3_r	AACCCTCACTAAAGGGAAGTTGGGTAACGCCAGGGTTT	
p#347_T3_f	CCCTTTAGTGAGGGTTAATTTAATTAAGGTACCGACTCG	
p#89_f	AAACCCGGCAAGCTGCCCGTGCCC	DIG probe for GFP
p#301_r	GAGCTCTTACTTGTACAGCTCGTCC	
p#466_f	AATTGCTATCTGGATGAAAGGCTAA	DIG probe for *CjChlI*
p#501_r	GAAGTTCTTCTTCTCTTTCAACTCTTTGC	
p#468_f	CTACGAAGGCTGTATCCAAGGACAATC	
p#469_r	CGGATCACCAGAAACCACTGT	Detection of mutation in *CjChlI*
p#648_r	GTGACAGGAAGTTCTTCTTCTCTTTCAACTCTT	

Supplementary Table S2 List of primers for cloning of CjU6 promoter

Plasmid	1st PCR		2nd PCR	
	Forward	Reverse	Forward	Reverse
pCjU6_#1	p#73	p#75_r	p#73	p#74_r
pCjU6_#2	p#76	p#77_r	p#76	p#74_r
pCjU6_#3	p#78	p#79_r	p#78	p#74_r
pCjU6_#4	p#76	p#107_r	p#76	p#74_r
pCjU6_#5	p#76	p#108_r	p#76	p#74_r
pCjU6_#6	p#76	p#109_r	p#76	p#74_r
pCjU6_#7	p#78	p#110_r	p#78	p#74_r
pCjU6_#8	p#78	p#111_r	p#78	p#74_r
pCjU6_#9	p#78	p#112_r	p#78	p#74_r
pCjU6_#10	p#96	p#97_r	p#96	p#74_r
pCjU6_#11	p#98	p#99_r	p#98	p#74_r

CjU6 promoter sequences (underlined sequences indicate U6 snRNA region)

CjU6 promoter #1

GGTCGAGAGATATGAAGAGCACCCCAGAAGCTGAGTTGGGGACATATTTGTAAACAGAAA
ACGAATCTTGCAAACAGAGTGATAAACAGAACAGTGGAGATGCGGGGTATCGAACCCCGT
ACCTCTCGCATGCAAAGCGAGCGCTCTACCATGTGAGCTACATCCCCGAATTGCTTACATC
CTTTCTCTTTCTTTTATCTTTCCACGAGAAAGGAAGCGCAGCGTTGTGAGCATTCGGTATGT
AGGGTAGGTTTAGGTTTCCTGANTGAAGATTCCCCTGGAACTGGAAANNCAATGGGCAAA
CATGCTGCTGATAGAGGCCTCTATAGCTCAGTGGTAGAGCGTCAGTCTTNGTAANACTGAA
GGTCCGTAGTTCGATCCTGCGTGGAGGCAAGGGTTAGGGTTGTTTGTTTGCTTGGTTAGCA
GCTGTGCTCGGTTGAAGTGTATTTTGCTTGGCAGGAGTTGAGGTGAGGTATCTGTAACATT
ACATTTTAGTTTTTTGATATAGCCTACTTTCTACTCTCTCGTGTCTCTACTCCGGAGGCGATG
GAAGGACGGACGGACAGACACGCGTGGCTTTAATCCCACATTTGTTACGCGTAAATGATAT
TAACACCACATATATTGTTTTGTACACATCTTCTT**TTCTCTTCGGAGACATCCGATAAAAT**
TGGAACGATACAGAGAAGATTAGCATGGCCCCTGCGCAAGGATGACACGCATAAATC
GAGAAATGGTCCAAATTTT

CjU6 promoter #2

GTGCGAGTCATATGTTGAGGTGAGGTATCTGTAACATTACGTTTTAGTTTTTTGATATAGCCT
ACTTTAATACCTTTAGCCTACTCTCTTGTGTCTCTACTCCCGAGGCGATGGAAGGACGGAC
GGACAGACAGGCGTGGCTTTAATCCCACATTTGTTACGCGTAAATGATATAGACACCTCGTA
TACTATTTTGTACGCCTGCTGTTGT**GTCTCTTCGGAGACATCCGATAAAATTGGAACGAT**
ACAGAGAAGATTAGCATGGCCCCTGCGCAAGGATGACACGCATAAATCGAGAAATGG
TCCAAATTTT

CjU6 promoter #3

CATTGTGGTCGTCGATATGTAGGATGGGTTTCCTGATCCAAGGTTCCCTGGCTTATCGTCAT

TGGCAAGAAATTGGGAACAGTGGGGTTCGATCCTGCATAGAGGCAGAGTGAAATTGTTTG

CTCGGTTAGCACCTCTGCACGCTTAAAGTGTATTTATCTGTTTTTGTTGGAGCAGGAGTTAA

GGTTTCTCTGTCACATTTTTCGTATACCAACTTCAATAAAACGGATACGTGTCTGTAGTCTC

TACTCCCGAGGCGATGGAAGGACGGACACGCGTGGCTTTGCTCCCACATTTAGTACGCATA

AATTGTATGAGCACTAAATATAACGTTACGGAGCCCGTTAATACCGCATAAATTAGATGTAC

GACATTTTTCATATACTTTAATACGGAGCCCTTTAATACCTCGGAGAACCGTGTCTCTAGTCT

CTAGTTCTACTCCCGAGGTGATGGGAGGACGAACACTTGTAACTTTACTCCCACATGCGCT

ACGCGTAAATGATGCAAGCACAACATATATTGTTTTGTACACGTATTTAAGT**GTCTCTTCG**

GAGACATCCGATAAAATTGGAACGATACAGAGAAGATTAGCATGGCCCCTGCGCAAG

GATGACACGCACAAATCGAGAAATGGTCCAAATTTT

CjU6 promoter #4

GTGCGAGTCATATGTTGAGGTGAGGTATCTGTAACATTACATTTTAGTTTTTTTGATATACTC

TACTTTAATACGTCTAGCCTACTCTATCGTGTCTCTACTCCCGAGGAGATGGAAGGAAGGAT

TGAAGGACACGCGTGGCTTTAATCCCACATTTGTTACGTGTAAATGATATTGACACCACATA

TATTGTTTTGTACGCTCCCACTAGT**GTTACTTCGGAGACATCCGATAAAATTGGAACGAT**

ACAGAGAAGAGTAGCATGGCCATTGAGCAAGGATGACACGCACAAATCGAGAAATG

GTCCAAATTTT

CjU6 promoter #5

GTGCGAGTCATATGtTGAGGTGAGGTATCTGTAACATTACATTTTAGTTTTTTGATATAGCCTA

CTTTCTACTCTCTCGTGTCTCTACTCCGGAGGCGATGGAAGGACGGACGGACAGACACGC

GTGGCTTTAATCCCACATTTGTTACGCGTAAATGATATTAACACCACATATATTGTTTTGTAC

ACATCTTCTT**<u>TTCTCTTCGGAGACATCCGATAAAATTGGAACGATACAGAGAAGATTAG</u>**

<u>CATGGCCCCTGCGCAAGGATGACACGCATAAATcGAGAAATGGTCCAAATTTT</u>

CjU6 promoter #6

GTGCGAGTCATATGTTGAGGTGAGGTATCTGTAACATTACATTTTAGTTTTTTGATATAGCCT
ACTTTAATACCTCTACCCTACTCTCTCGTGTCTCTACTCCCGAGGCGATGGAAGGACGGAC
GGACAGACAGGCGTGGCTTTAATCCCACATTTGTTACGCGTAAATGATATTGACACCTCGTA
TACTATTTCGTACGCCTCTTGTTGT**<u>GTCTCTTCGGAGACATCCGATAAAATTGGAACGAT</u>**
<u>ACAGAGAAGATTAGCATGGCCCCTGCGCAAGGATGACACGCATAAATCGAGAAATGG</u>
<u>TCCAAATTTT</u>

CjU6 promoter #7

CATTGTGGTCGTCGATATGTAGGATGGGTTTCCTGATCCAAGGTTCCCTGACTTATCGTCAT

TGGCAAGAAATTGGGAGCAATGGGGTTCGATCCTGCGTGGAGGCAGAGTGAAATTGTTTG

CTCGGTTAGCACCTGCGCACGCTTAAAGTGTATTTATCTGTTTTCGTTGGAACAGGAGTTAA

GGTTTCTCTGTGACATTTTTCGTATACCTACTTCAATAAAACGGATAAACGTGTCTGTAGTC

TCTACTCCCGAGGCGACGGAAGGACGGACACGCGTGGCTTTCCTCCCACATTCGCTACGC

ATAAATTATATGGGCACTACATATAACGTTGTGTACGCGTAAATTAAT**<u>GTCTCTTCGGAGAC</u>**

<u>ATCCGATAAAATTGGAACGATACAGAGAAGATTAGCATGGCCCCTGCGCAAGGATGA</u>

<u>CACGCACAAATCGAGAAATGGTCCAAATTTT</u>

CjU6 promoter #8

CATTGTGGTCGTCGATATGTAGGGTAGGTTTCCTGATCCAAGGTTCCCTGTTTTATCATCATT
GGCTAGAAATTGGGAACAATGGGGATCGATCCTGCGTGGAGGCAGAGTGGTTTGTTTGCT
CAGTTTGCAGCTGTACACGCGTGATGTGTATTTAACTGTTTTGCTTGGACCAGGAGTTGAA
GTTTTTCTGTGACATTTTTCATATACTTCAATACCTCGGATAACCGTGTATCTAGTCTCTACTC
CCGAGGCGATGGAAGGACGGACACGCGTGGCTTTAATCCCACATTCGCTACACGTAAAGG
GTATAAGCACGAAATATACTGTTTACGATTATGGTA**GTGTCTCTTTGGAGACATCCGACA
AAATTGGAACGATACAGAGAAGATTAGCATGGCCCCTGCGCAAGGATGACACGCATA
AATCGAGAAATGGTCCAAATTTT**

CjU6 promoter #9

CATTGTGGTCGTCGATATGTAGGATGGGTTTCCTGATCCAAGGTTCCCTGGCTTATCGTCAT
TGGCAAGAAATTGGGAACAATGGGGTTCGATCCTGCGTGGAGTCAGGGTCAAATTGTTTG
CTCGGTTAGCACCTGCGCACGCTTAAAGTGTATTTATCTGTTTTCATTGGAGCAGGAGTTAA
GGTTTCTCTGTGACATTTTTCGTATACCTAGTTCAATAACTCGGATAACCGTGCCTGTAGTCT
CTATTCTCGAGGCGATGGAAGGACAGACACGCGTGGCTTTACTCCCACATTCACTAAGCGT
AAATGATATGAGCACTACATATACCGTTTTGTATTGACAGTTGAAT**GTCTCTTCGGAGACA
TCCGATAAAATTGGAACGATACAGAGAAGGATTAGCATGGCCCCTGCGCAAGGATGA
CACGCATAAATCGAGAAATGGTCCAAATTTT**

CjU6 promoter #10

AGTGGAGTAGCAGAGACAGAGACAGGAATTGGGGGAAAAGCTGGAACCTAAATCGAACA
TAAACAAAACTAAAATTTGAAACCGGACTCTATATTTCCTAGAAGATGACGACATGAATGG
ATTCCTATAGTGACTTCTAAAACTGTACCAGTCCGCAGTAATAAATGCGGTTTCCTCGCACT

TATAGCAGAAGCAGGAGCTGGAATTCACAGTAATAATATATATTTTATTACTTCCTTCCTCTG

CGTATAAATTGGATTCGTCTCTCTCCGTGCAATGCAAGGACGGCTGGTGTCGCCTAAGGTA

CACTCTATTTACTCCCACATGCTACGTACGTAGATAATTCAAACGCTTTATATTATGTTTTGTA

CAAATTGTCAAT**GTCTCTTCGGAGACATCCGATAAAATTGGAACGATACAGAGAAGAT**

TAGCATGGCCCCTGCGCAAGGATGACACGCANAAATCGAGAAATGGTCCAAATTTT

CjU6 promoter #11

GTGCGAGACATATGTTGCAGATAAAATGTTTGAAAATTGATGGAAGAAAAAGTTGCCTGCT

AAGCTGAGAAGTGTGCCGGCAGTTTGAGATACGGTAGACAATGAAACCGCGGGCAGTGC

GACCATCAAACCGGCGAATTTAAAGCCCAAAATCGGTGGGCATTCCGAGAATTTGACGGC

ATCTAGTCGGTTATCTGATTTTGTAAAGCAAGGGCTGCTGGAGTAGTCTAAAGTTCCTTCAT

GACAGGTTAAGTCGAAGCCACTTCTGTACCCGTTGCGCGACGGGGTTGTTACCTTAATACC

ACATTGTATACAACTCTAAATAAGAGACTTTAAATATAGAGAGCTAGTGACCTTGAAAT**GTC**

TCTTCGGAGACATCCGATAAAATTGGAACGATACAGAGAAGATTAGCATGGCCCCTG

CGCAAGGATGACACGCACAAATCGAGAAATGGTCCAAATTTT

CjEF1αpromoter sequence

GAGTACATATTTCTGACGAGGAAATTCACTTTAACTTAAAACTCTTCCATTAACCAATCAGT
GGTCAAAGTCAACACGCATTAATTATTGGCCGCACTATTCTACGCATGCAAGTGGTATTCAT
AATAGATTTGAAAACAAGTACATACATATATTTTTATTAATTAAAAATAGATTTATCTTTTTT
TATGCATTAAAAATATATTTAGAAAGCCTATTAGGCGGTGCATTAATAATTGGCTTTTAAAAA
AACACCAAGTCTAATAATAAAACCTGAATTATATAAATTTTACAAAATTAAAAGTCGATTAA
AATTGTTTTCTAAGTGGTGGTATATTAGGTATAGGATTATTTTAATATAAGAAATTTCTAGTTG
CATTATGCACTTAAATGGAACCCATTATAGATTGCCATAATAAAAATAAATTAAAACAGCCA
AGAATAAAGTTATTAAAAAAATTAAGAATAAAATTATTAAAAAAAAAAGAGGAAAAAAAA
GCCAAGAATAAGAAAACATAAGGTGGATACCTAATTTAAGCGGAGATTAAAAATTGTCGGT
TTTGCCGGCCTGTCGGAAGGAACGAATTTCTTTTTATGCTTTCACAAAAGGTTAAAAGATT
CTTCTTCGAGATTAGCCCCGGTCAATCTCAGCCGTCCGTTGTAAAATTGGACGGTTGACAC
AAGGACTTAATCTCAACCGTTGAAATAAATATCGGACGGCGAGGAAAAGTAACCTAATTTC
TAAGTGGAAGGGTTTGAGGCAAAAAGGCTATATCTTTGCTTTGCAGCGTTAAGAAGGGTCT
CTTTAAGCGAGGCAATTTTTCTTCTTCCTTCGCCGCTGCGGATACTCCCAACAAGCTATTCT
TCGATCTGCCAGGTGCGTCTGTATTAAGGTCTGGTACCTTGCTTGTTTGCTTGTTTGCTGTA
CTTTTATTTGCTTCGAGGTATGTCTTTTGATAGAAATGGCTGTTTTAGTTGATGTTTGGTAAG
TCAAGGGGTTTGATTGGTTTCCGTTAGCGGATGTGCCGTTGTAGATCTTAATGGATCTTATTT
AAGCTTCTGTATGGTTGGGTTATGAGGTGGTTGAGGTATGATTGCGGATTATGTTTTCGATG
TATTTTGCTCTTGGTGAGGGTTTGTAATGTTCATTTTGTACATTGATCTGTTAGTTTATGACT
CTGTTCTTTTACTGTATGCTCGATCTGGTTGTTTTTATTGTTTTTTGTGATTTTTGTCTAGGGT
TTAAGGTTTTTCTTTTTGTTAGTTTTTGGCTTTGTGTTCTTATTGTATGCTCGATCTGGTTGAT
TTTTAAGGTTTTTGTGAGCTTTAGTCCAGGGTTTAAGGTTTTCCTCTTTGTTCCGGCTTGTC
AGAGAAGATCTGGATCTTTAGGAAAAATAGATGAATTTGTTGCCTTGATTTAATTTTATTCTC
CAAAGATCGGGCTTTGACTCGTTGCAACTCTCTCATCTGAGGAAATTGCAATTTCTGTTTG
GTTGTTCTTGTTGTCATAGTCTATTTTATTCTTCAAAGTAATCACTTGTGGCCGATTTACTAC
CCCTTTTGAGTAGTTTTATGATGACATTTAAGTTGCTCCATCATTGCTTTTATTTTTGAAAAC
TATTTAACTTCTTGTTTTAATCAGGCACTTAAACGAATAGTATTATGATGACATTTAGGCTGT
TCTAATACGTGGTTTACGATGGTCCTTATAGGTATCCAAGAAGATTACTGTTGTCTAATAATG
TTCTGTTTTTCTCTGGTGCTTGTTTATTTATTAAATCTGTTGCCTGAGATTTATCCTGCAATAT
GCAGAAGTTTGCTCTGTTTTTAAGCATTGTCCGGAGTAATTGTTTTCAAATTTATCTATTATC
TTATTAAACTATAGTTTTCTTCTATTTCTTAGAGGCGTTCCTTGTTTGGAATTTATTCTTTTTG
TTATTGTTTTAATATTTAGATTATCAATTTTCTCCTTTTTGCTGTTTTAGGTATCCTCTGTTTTC
TCATTTGATTTCTTTGTGTCGTTTTATTATACTATTTTATTAAATCTAATCTTCATTAGCATCTC
TTTTAAAATAAATGCAACTCACAGACATTAATTAAATCGTCATTTCTATGAAAGTCTAGCTG

GGCGTGATGTAACAATGTCTATTGATTTCTTGATACCTCGTTGCCTGTTTTAATTTCTGAGTT
TGATATTTCTCCAAAGAGAACCTTACTTGTTTTCTTCATGTAACAAATGTTTATTGCAATATT
GAATTCTGAGATTGATATTTCTTCAAAGAGTGACTTGGTTTCTTCCACCTGTTTCAGTCCAG
GGGTGTATTTTTTTACTCCGTCTTTTAATCACTCAATTTTTTCTGTCCTCTCTGCTGCTCCTAT
TATATAACTAGGGCGTGGACTATTTGTGGTGTTTATTTGATGAGTATTATGTATTTTTGGTAGT
CTTGAGAGGGGTCATGCAAACCTAACTGTGTTCTATTATTAGTAGTTGGAGCTATACTCTTAT
TTTTTGTAAATAGTATTTATTAAATATCATGTTCAGTGTCTCTTCTCTTTGAGTTAATTCCCAC
TTGTATCATGGATTATTTTATATGTATGTGACTTGACAATCATAATTATTTTCATGTGACTTGT
GGGAAGAATCAATTTTTAAAAGTTACTTGACTGATAAACTTTTGGTTACAGTGCTTGATACT
TTAGGTTCATGTTGTTTTTTTAGTAAAGGTGATTTATAGTCTCTAGTTGGTATGATGCCTCTAT
GTTAGCAGCTTTAGATATTCATGTGGAGCTCTATTTGCTGTCAGCGTTTGGGATTTATGTCGG
GGTTTTCAAAACTCCTAGGTTTTAGGAAGGCACACACAAATCTTTAAAGAGGCATTGACA
AAAGCTTATTTTTTGTTACTGAATTCTTTAGAGGTTATTTAATCACAAGAAATGCTGTGGGT
GATATTTAACCTCGATGGCTTTGCTTTACTTTTGCAGTTTTTGACTACAAGAAACC

References

1. Nagaya, S., Kawamura, K., Shinmyo, A. & Kato, K. The HSP terminator of *Arabidopsis thaliana* increases gene expression in plant cells. *Plant Cell Physiol.* **51**, 328–332 (2010).
2. Shimada, S., Otsuki, H. & Sakuta, M. Transcriptional control of anthocyanin biosynthetic genes in the Caryophyllales. *J. Exp. Bot.* **58**, 957–967 (2007).
3. Liu, Y.-G., Mitsukawa, N., Oosumi, T. & Whittier, R. F. Efficient isolation and mapping of *Arabidopsis thaliana* T-DNA insert junctions by thermal asymmetric interlaced PCR. *Plant Journal* **8**, 457–463 (1995).
4. Liu, Y.-G. & Whittier, R. F. Thermal asymmetric interlaced PCR: automatable amplification and sequencing of insert end fragments from P1 and YAC clones for chromosome walking. *Genomics* **25**, 674–681 (1995).
5. Mitsuhara, I. *et al.* Efficient promoter cassettes for enhanced expression of foreign genes in dicotyledonous and monocotyledonous plants. *Plant Cell Physiol.* **37**, 49–59 (1996).
6. Christensen, A. H. & Quail, P. H. Ubiquitin promoter-based vectors for high-level expression of selectable and/or screenable marker genes in monocotyledonous plants. *Transgenic Res.* **5**, 213–218 (1996).
7. Kawalleck, P., Somssich, I. E., Feldbrügge, M., Hahlbrock, K. & Weisshaar, B. Polyubiquitin gene expression and structural properties of the *ubi*4-2 gene in *Petroselinum crispum*. *Plant Mol. Biol.* **21**, 673–684 (1993).
8. Konagaya, K. I., Kurita, M. & Taniguchi, T. High-efficiency *Agrobacterium*-mediated transformation of *Cryptomeria japonica* D. Don by co-cultivation on filter paper wicks followed by meropenem treatment to eliminate *Agrobacterium*. *Plant Biotechnol.* **30**, 523–528 (2013).

9. Doench, J. G. *et al.* Rational design of highly active sgRNAs for CRISPR-Cas9–mediated gene inactivation. *Nat. Biotechnol.* **32**, 1262–7 (2014).

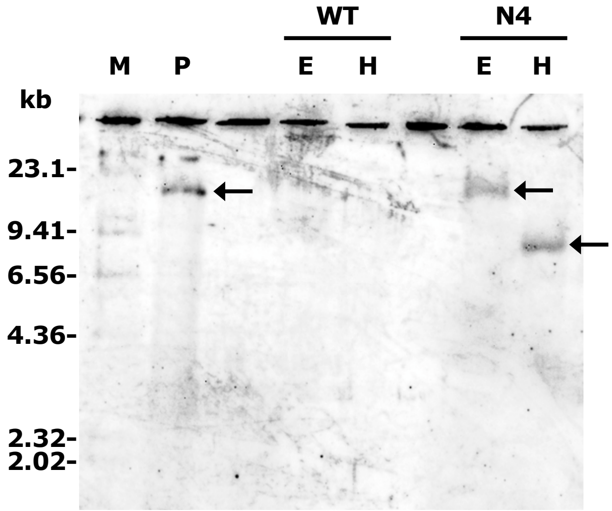

Supplementary Figure S1. Southern blot analysis of *Eco*RV- and *Hin*dIII-digested genomic DNAs from embryogenic tissue (ET) of wild-type line #13-8-12 (WT) and GFP-expressing line (N4). The blot was probed with a fragment internal to GFP. Arrows indicate probe hybridization with complementary DNA sequence. The number of bands refers to the copy number of target DNA sequence in the genome. Lane M; DIG-labeled λ-*Hin*dIII, lane P; pZmUbi-GFP-Dt vector as a positive control template, E; *Eco*RV, H; *Hin*dIII. Full-length blot image is included in Supplementary Figure S8a.

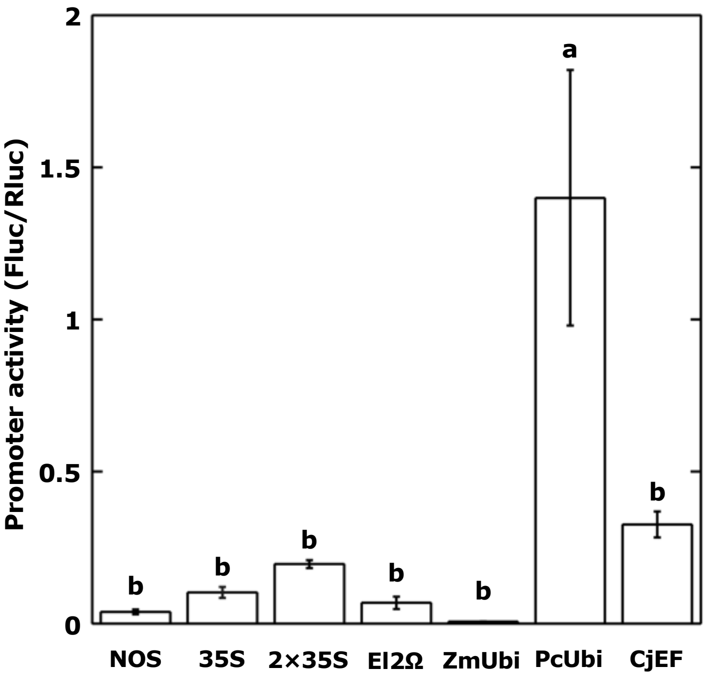

Supplementary Figure S2. Comparison of promoter activity in the embryogenic tissue (ET) of *Cryptomeria japonica* using the dual luciferase assay. Analyses were conducted as a one-way ANOVA and means were separated by Tukey's HSD. Values labeled with different letters differ significantly ($P < 0.05$). NOS, nopaline synthase promoter; 35S, promoter of the 35S gene of the cauliflower mosaic virus; 2×35S, tandem 35S; El2Ω, 2×35S with omega sequence; ZmUbi, ubiquitin promoter from *Zea mays*; PcUbi, polyubiquitin promoter from *Petroselinum crispum*; CjEF1α, elongation factor 1α promoter from *C. japonica.*

```
                                       USE                            TATA
#11              GGGGTTGTTAC-----CTTAATACCACATTGTATA--CAACTCTAA-ATAAGAGACTTTAAATATAGAGA
OsU6             GGGAGGAACAG-----TTTAGTACCACATTGCCCAGCTAACTCGAACGCGACCAACTTATAAACCCGCGC
#7               ACGCGTGGC-------TTTCCTCCCACATTCGCTACGCATAAATTATATGGGC-ACTACATATAACGTTG
#9               ACGCGTGGC-------TTTACTCCCACATTCACTAAGCGTAAATGATATGAGC-ACTACATATACCGTTT
#8               ACGCGTGGC-------TTTAATCCCACATTCGCTACACGTAAAGGGTATAAGC-ACGAAATATACTGTT-
#1               ACGCGTGGC-------TTTAATCCCACATTTGTTACGCGTAAATGATATTAAC-ACCACATATATTGTTT
#5               ACGCGTGGC-------TTTAATCCCACATTTGTTACGCGTAAATGATATTAAC-ACCACATATATTGTTT
#2               AGGCGTGGC-------TTTAATCCCACATTTGTTACGCGTAAATGATATAGAC-ACCTCGTATACTATTT
#6               AGGCGTGGC-------TTTAATCCCACATTTGTTACGCGTAAATGATATTGAC-ACCTCGTATACTATTT
#4               ACGCGTGGC-------TTTAATCCCACATTTGTTACGTGTAAATGATATTGAC-ACCACATATATTGTTT
#3               ACTTGTAAC-------TTTACTCCCACATGCGCTACGCGTAAATGATGCAAGC-ACAACATATATTGTTT
#10              CTAAGGTACACTCTA-TTTACTCCCACATGCTACGTACGTAGATAATTCAAAC-GCTTTATATTATGTTT
AtU6             GTTGAAAACAATCTTCAAAAGTCCCACATCGCTTAGATAAGAA-AACGAAGCTGAGTTTATATACAGCTA
                                      * ******                                *
                                +1
                                 .
#11              GCTAGTGACCTTGAAATGTCTCTTCGGAGACATCCGATAAAATTGGAACGATACAGAGAAGATTAGCATG
OsU6             GCTGTCG--CTTGTGTTGTCTCTTCGGAGACATCCGATAAAATTGGAACGATACAGAGAAGATTAGCATG
#7               TGTACGCGTAAATTAATGTCTCTTCGGAGACATCCGATAAAATTGGAACGATACAGAGAAGATTAGCATG
#9               TGTATTGACAGTTGAATGTCTCTTCGGAGACATCCGATAAAATTGGAACGATACAGAGAAGATTAGCATG
#8               --TACGATTATGGTAGTGTCTCTTTGGAGACATCCGACAAAATTGGAACGATACAGAGAAGATTAGCATG
#1               TGTACACATCTTCTTTT--CTCTTCGGAGACATCCGATAAAATTGGAACGATACAGAGAAGATTAGCATG
#5               TGTACACATCTTCTTTT--CTCTTCGGAGACATCCGATAAAATTGGAACGATACAGAGAAGATTAGCATG
#2               TGTACGCCTGCTGTTGTGTCTCTTCGGAGACATCCGATAAAATTGGAACGATACAGAGAAGATTAGCATG
#6               CGTACGCCTCTTGTTGTGTCTCTTCGGAGACATCCGATAAAATTGGAACGATACAGAGAAGATTAGCATG
#4               TGTACGCTCCCACTAGTGTTACTTCGGAGACATCCGATAAAATTGGAACGATACAGAGAAGAGTAGCATG
#3               TGTACACGTATTTAAGTGTCTCTTCGGAGACATCCGATAAAATTGGAACGATACAGAGAAGATTAGCATG
#10              TGTACAAAT-TGTCAATGTCTCTTCGGAGACATCCGATAAAATTGGAACGATACAGAGAAGATTAGCATG
AtU6             GAGTCGAAGTAGTGATTGTCCCTTCGGGGACATCCGATAAAATTGGAACGATACAGAGAAGATTAGCATG
                                 *    *** ** ********* ************************ *******

#11              GCCCCTGCGCAAGGATGACACGCACAAATCGAGAAATGGTCCAAATTTT
OsU6             GCCCCTGCGCAAGGATGACACGCACAAATCGAGAAATGGTCCAAATTTT
#7               GCCCCTGCGCAAGGATGACACGCACAAATCGAGAAATGGTCCAAATTTT
#9               GCCCCTGCGCAAGGATGACACGCATAAATCGAGAAATGGTCCAAATTTT
#8               GCCCCTGCGCAAGGATGACACGCATAAATCGAGAAATGGTCCAAATTTT
#1               GCCCCTGCGCAAGGATGACACGCATAAATCGAGAAATGGTCCAAATTTT
#5               GCCCCTGCGCAAGGATGACACGCATAAATCGAGAAATGGTCCAAATTTT
#2               GCCCCTGCGCAAGGATGACACGCATAAATCGAGAAATGGTCCAAATTTT
#6               GCCCCTGCGCAAGGATGACACGCATAAATCGAGAAATGGTCCAAATTTT
#4               GCCATTGAGCAAGGATGACACGCACAAATCGAGAAATGGTCCAAATTTT
#3               GCCCCTGCGCAAGGATGACACGCACAAATCGAGAAATGGTCCAAATTTT
#10              GCCCCTGCGCAAGGATGACACGCACAAATCGAGAAATGGTCCAAATTTT
AtU6             GCCCCTGCGCAAGGATGACACGCATAAATCGAGAAATGGTCCAAATTTT
                 ***  ** **************** ************************
```

Supplementary Figure S3. Sequence alignment of 11 *Cryptomeria japonica* U6 snRNA genes and their proximal promoter regions with those from *Arabidopsis thaliana* and *Oryza sativa.* Asterisks indicate conserved nucleotides. Upstream sequence element (USE) is boxed in green. TATA-box is indicated in red letters. The putative transcription start site is shown as "+1."

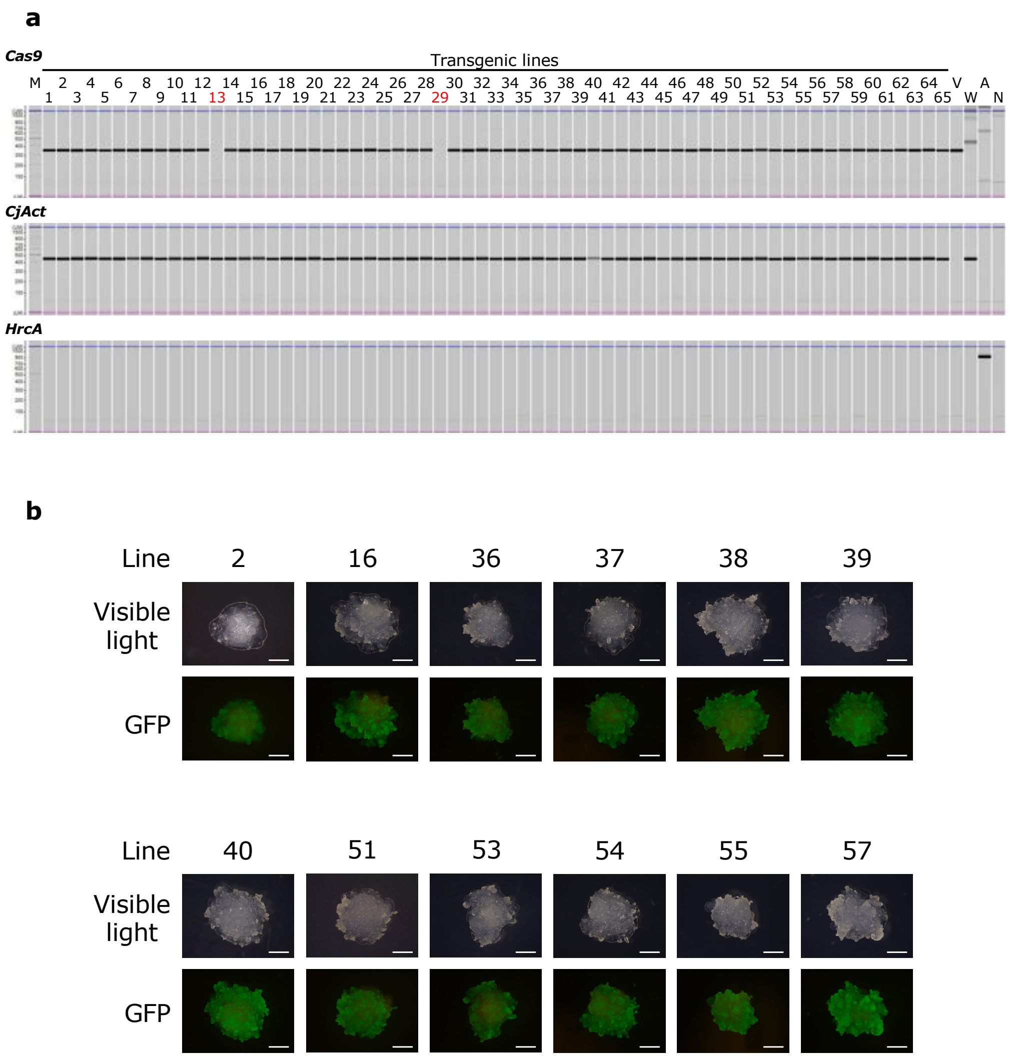

Supplementary Figure S4. Molecular analysis of negative control lines. (a) Genomic PCR analysis of N4 transgenic lines containing the pZK_FFCas9 transgenes without the gRNA expression cassette. *HrcA* is used for monitoring *Agrobacterium* contamination. Transgenic lines numbered in red represent escape lines. V, pZK_FFCas9 vector plasmid; W, WT genomic DNA (ET line #13-8-12); A, *Agrobacterium* genomic DNA; N, mock (no template). (b) GFP fluorescence in 12 transgenic lines. Bars, 2 mm.

```
GCCGAGGTGAAGTTCGAGGGCGACACCCTGGTGAACCGCATCGAGCTGAAGGGCATCGACTTCAAGGAGGACGGCAACATCCTGGGGCACAAGCTGGAGTA  GFP

GCCGAGGTGAAGTTCGAGGGCGACACCCTGGTGAACCGCATCGAGCT-AAGGGCATCGACTTCAAGGAGGACGGCAACATCCTGGGGCACAAGCTGGAGTA  d1   ×1
GCCGAGGTGAAGTTCGAGGGCGACACCCTGGTGAACCGCATCGA--TGAAGGGCATCGACTTCAAGGAGGACGGCAACATCCTGGGGCACAAGCTGGAGTA  d2   ×2
GCCGAGGTGAAGTTCGAGGGCGACACCCTGGTGAACCGCATCG---TGAAGGGCATCGACTTCAAGGAGGACGGCAACATCCTGGGGCACAAGCTGGAGTA  d3   ×2
GCCGAGGTGAAGTTCGAGGGCGACACCCTGGTGAACCGCATCGAG---AAGGGCATCGACTTCAAGGAGGACGGCAACATCCTGGGGCACAAGCTGGAGTA  d3   ×1
GCCGAGGTGAAGTTCGAGGGCGACACCCTGGTGAACCGCAT------GAAGGGCATCGACTTCAAGGAGGACGGCAACATCCTGGGGCACAAGCTGGAGTA  d6   ×2
GCCGAGGTGAAGTTCGAGGGCGACACCCTGGTGAA---------------GGGCATCGACTTCAAGGAGGACGGCAACATCCTGGGGCACAAGCTGGAGTA  d15  ×6
GCCGAGGTGAAGTTCGAGGGCGACACCCTGGTGAACCGCATCGA---------------CTTCAAGGAGGACGGCAACATCCTGGGGCACAAGCTGGAGTA  d15  ×10
GCCGAGGTGAAGTTCGAGGGCGACACCCTGGTGAACCGCATCGAGCT---------------CAAGGAGGACGGCAACATCCTGGGGCACAAGCTGGAGTA  d15  ×1
GCCGAGGTGAAGTTCGAGGGCGACACCCTGGTctACatCcTgGgGCa---------------CAAGctGGAgtaCAACATCCTGGGGCACAAGCTGGAGTA  d15r ×1
GCCGAGGTGAAGTTCGAGGGCGACACCCTGGTGA---------------------TCGACTTCAAGGAGGACGGCAACATCCTGGGGCACAAGCTGGAGTA  d21  ×1
GCCGAGGTGAAGTTCGAGGGCGACACCCTGGTGA-------------------------CTTCAAGGAGGACGGCAACATCCTGGGGCACAAGCTGGAGTA  d25  ×1
GCCGAGGTGAAGTTCGAGGGCGACACCCTGGTGAACCGCATC---------------------AAGGAGGACGGCAACATCCTGGGGCACAAGCTGGAGTA  d21  ×1
GCCGAGGTGAAGTTCGAGGGCGACACCCTGGTGAACCGCATCGA---------------------------CcGCAACATCCTGGGGCACAAGCTGGAGTA  d27r ×1
GCCGAGGTGAAGTTCGAGGGCGACACCCTGGTGAACCGCA------------------------------------ACATCCTGGGGCACAAGCTGGAGTA  d36  ×3
GCCGAGGTGAAGTTCGAGGGCGACACCCTGGTGAACCGCATCGAGCa-AA-------GACccCAAGGAGGACGGCAACATCCTGGGGCACAAGCTGGAGTA  d8r  ×1
GCCGAGG----(13)-----CGACACCCTGGTGAACCGCATCGA------(15)-----CTTCAAGGAGGACGGCAACATCCTGGGGCACAAGCTGGAGTA  d28  ×1
GCCGAGGTGAAGTTCGAGGGCGACACCCTGGTGAACCGCATCGAG------------------------------------------------------TA  d54  ×1
GCCGAGGTGAAGTTCGAGGGCGACACCCTGGTa------------------------------------------------------CACAAGCTGGgGTA  d54r ×1
GCCGAGGTGAAGTTCGAGGGCGACACCCTGGTGAACCGCATC------------------------------------ATCCTGGGGCACAAGCTGGAGTA  d36  ×1
GCCGAGGTGAAGTTCGAGGGCGACACCCTGGTGAACCGCATCGAG------------------------------------------CACAAGCTGGAGTA  d42  ×1
GCCGAGGTGAAGTTCGAGGGCGACACCCTGGTGAAC------------------------------------------ATCCTGGGGCACAAGCTGGAGTA  d42  ×1
GCCGAGGTGAAGTTCGAGGGCGACA---------------------------------------------------ACATCCTGGGGCACAAGCTGGAGTA  d51  ×1
GCCGAGGTGAAGTTCGAGGGC------------------------------------------------------AACATCCTGGGGCACAAGCTGGAGTA  d54  ×1
GC----------------------------------------------------------TTCAAGGAGGACGGCAACATCCTGGGGCACAAGCTGGAGTA  d58  ×1
GCCGAGGTGAAGTTCGAGGGCGACACCCTGGTGAACCGCATCGAGCTTGAAGGGCATCGACTTCAAGGAGGACGGCAACATCCTGGGGCACAAGCTGGAGT  i1   ×5
GCCGAGGTGAAGTTCGAGGGCGACACCCTGGTGAACCGCATCGAGGCTGAAGGGCATCGACTTCAAGGAGGACGGCAACATCCTGGGGCACAAGCTGGAGT  i1   ×1
GCCGAGGTGAAGTTCGAGGGCGACACCCTGGTGAACCGCAATCGAGCTGAAGGGCATCGACTTCAAGGAGGACGGCAACATCCTGGGGCACAAGCTGGAGT  i1   ×1
GCCGAGGTGAAGTTCGAGGGCGACACCCTGGTGAACCGCATCGAGCCACCCTGGTGATGTTGCTGAAGGGCATCGACTTCAAGGAGGACGGCAACATCCTG  i17  ×1
GCCGAGGTGAAGTTCGAGGGCGACACCCTGGTGAACCGCATCGAGCAAAGACCCCAACGAGAAGCGCGATCACATCACCTGAAGGGCATCGACTTCAAGGA  i33  ×1
```

Supplementary Figure S5a. Mutation patterns in GFP target #1. Target and PAM sequences are marked in blue and green, respectively. The sequence of wild-type GFP is shown in the "GFP" column. Numbers on the right after "×" indicate the number of sequenced clones. Yellow boxes indicate microhomologies between the target sequence. A deletion is denoted by "–." Letters in red and lower-case pink indicate inserted and replaced bases, respectively. d#, i#, and r# denote the number of bp deleted, inserted, and replaced at the target site, respectively.

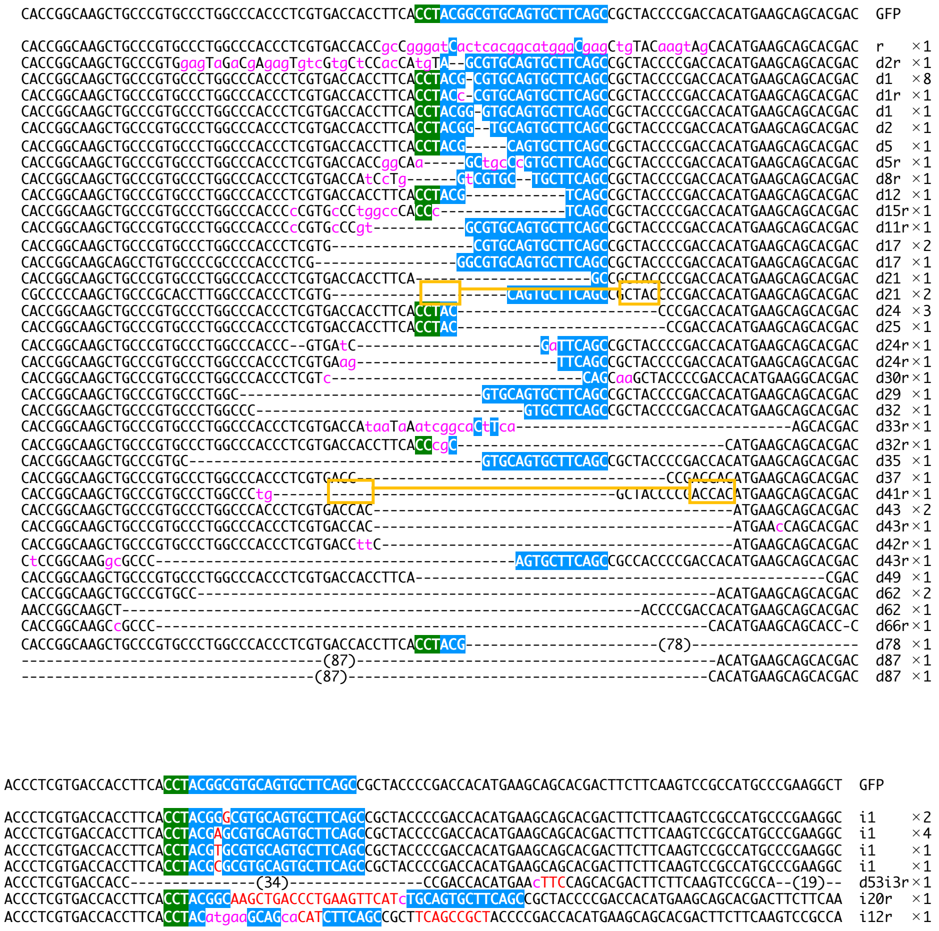

Supplementary Figure S5b. Mutation patterns in GFP target #2_rev. Target and PAM sequences are marked in blue and green, respectively. The sequence of wild-type GFP is shown in the "GFP" column. Numbers on the right after "×" indicate the number of sequenced clones. Yellow boxes indicate microhomologies between the target sequence. A deletion is denoted by "−." Letters in red and lower-case pink indicate inserted and replaced bases, respectively. d#, i#, and r# denote the number of bp deleted, inserted, and replaced at the target site, respectively.

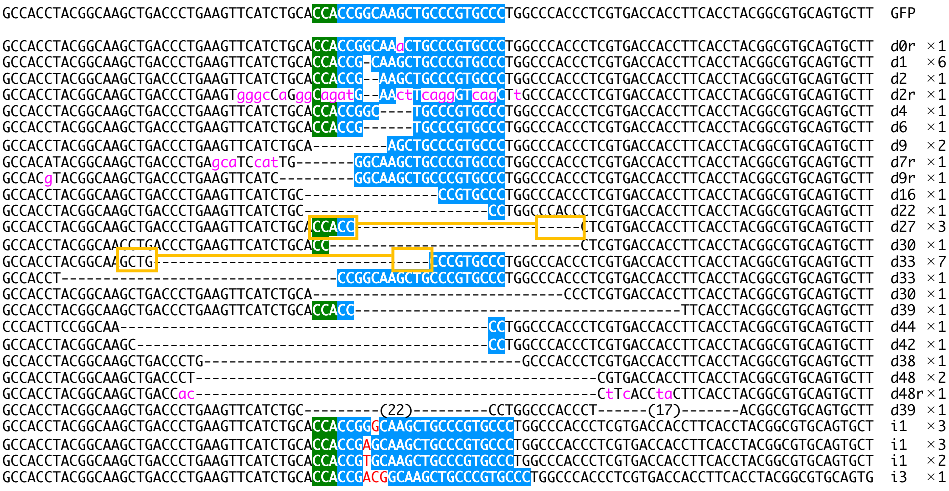

Supplementary Figure S5c. Mutation patterns in GFP target #3_rev. Target and PAM sequences are marked in blue and green, respectively. The sequence of wild-type GFP is shown in the "GFP" column. Numbers on the right after "×" indicate the number of sequenced clones. Yellow boxes indicate microhomologies between the target sequence. A deletion is denoted by "–." Letters in red and lower-case pink indicate inserted and replaced bases, respectively. d#, i#, and r# denote the number of bp deleted, inserted, and replaced at the target site, respectively.

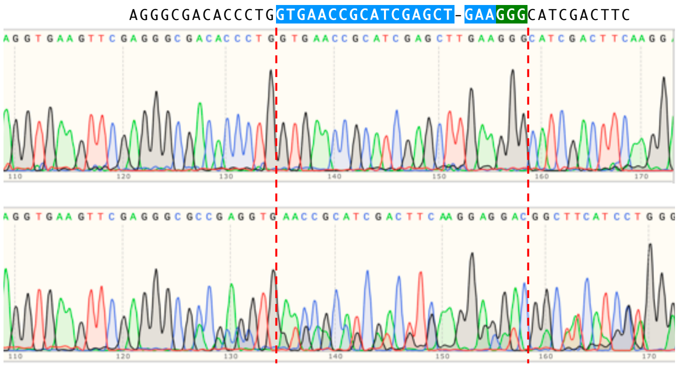

Supplementary Figure S6. Sequence chromatograms in cotyledonary embryos from line #47-2. Target and PAM sequences are marked in blue and green, respectively. Chromatograms from an individual having i1 mutation (upper panel) and multiple modification patterns (lower panel) are indicated, respectively.

a

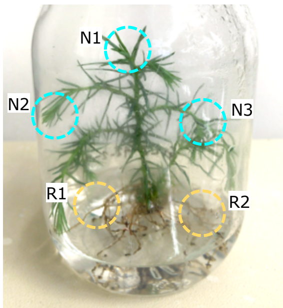

b

Line	Position	Sequence of target site (5’-3’)	Mutation	No. of plantlets
GFP	-	AGGGCGACACCCTGGTGAACCGCATCGAGCTGAAGGGCATCGACTTC	-	-
#42-2	All	AGGGCGACACCCTGGTGAACCGCATCGAGCTGAAGGGCATCGACTTC	-	1
	All	AGGGCGACACCCTGGTGAACCGCATCGA---------------CTTC	d15	6
	N1-N3<br>R1, R2	AGGGCGACACCCTGGTGAACCGCATCGAGCTGAAGGGCATCGACTTC<br>AGGGC-----------------(54)---------------------	-<br>d54	2
	N1-N3<br>R1, R2	AGGGCGACACCCTGGTGAACCGCATCGAGCTGAAGGGCATCGACTTC<br>AGGGCGACACCCTGGTGAACCGCATCGAGCTTGAAGGGCATCGACTT	-<br>i1	1
	N1-N3<br>R1<br>R2	AGGGCGACACCCTGGTGAACCGCATCGAGCTGAAGGGCATCGACTTC<br>AGGGCGACACCCTGGTGAACCGCATCGA---------------CTTC<br>AGGGCGACACCCTGGTGAACTTCA---AGaTccgccaCtcgaACTTC	-<br>d15<br>d3r	1
#31-2	All	AG---------------------(54)--------------------	d54	10
#11	All	AGGGCGACACCCTGGTGAACCGCATCGA---------------CTTC	d15	10
#18	All	AGGGCGACACCCTGGTGAACCt--------------GCATCGACTTC	d14r	5
	All	AGGGC---------------------------------ATCGACTTC	d33	1
	All	AGGGCGACACCCTGGTGAACCGCATCGA---------------CTTC	d15	1
	All	AGGGCGACACCCTGGTGAACCGCATC-------(39)----------	d39	1
	N1-N3<br>R1<br>R2	AGGGCGACACCCTGGTGAACCGCATCGAGCTGAAGGGCATCGACTTC<br>-------------------(140)-----------------------<br>AGGGCGACACCCTGGTGAACCGCATCGAGCT---------------C	-<br>d140<br>d15	1
	N1-N3<br>R1<br>R2	AGGGCGACACCCTGGTGAACCGCATCGAGCTGAAGGGCATCGACTTC<br>-------------------(81)---------------------TTC<br>AGGGCGACACCCTGGTGAACCGCATCGA--TGAAGGGCATCGACTTC	-<br>d81<br>d2	1

Supplementary Figure S7. Mutation patterns in GFP knock-out plantlets. (**a**) Sampling position in the plantlet. (**b**) Table showing detailed mutation pattern at each position. Target and PAM sequences are marked in blue and green, respectively. Sequence of wild-type GFP is shown in the “GFP” column. A deletion is denoted by “–.” Letters in red and lower-case pink indicate inserted and replaced bases, respectively. d# and i# denote the number of bp deleted and inserted at the target site, respectively. We extracted genomic DNAs from the needle or root segments and determined the nucleotide sequences by direct sequencing.

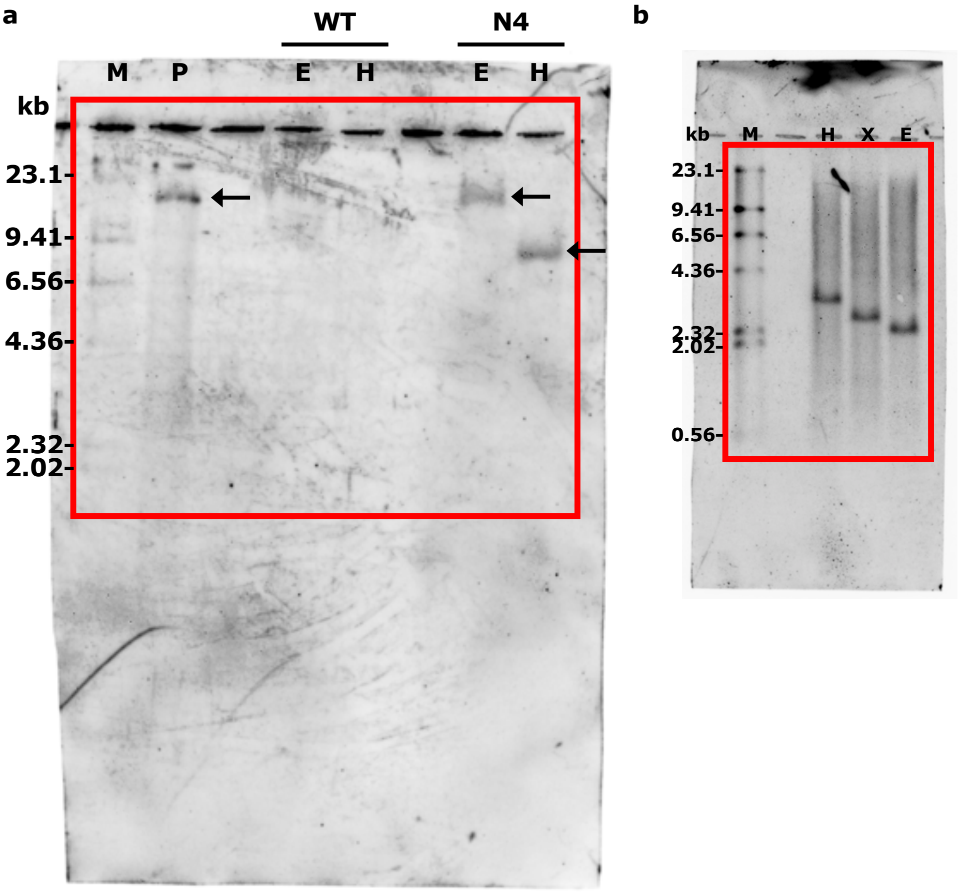

Supplementary Figure S8. Full-length images of the blots presented in Supplementary Figure S1 (**a**) and Figure 5b (**b**). Red boxes indicate cropped regions.
